# rFVIIIa-platelet binding enhances platelet procoagulant activity independently of thrombin generation

**DOI:** 10.1016/j.bvth.2025.100132

**Published:** 2025-12-10

**Authors:** Anja Strebel, Sebastian Lickert, Robert Klamroth, Viola Vogel, Fabrizio A. Pennacchio

**Affiliations:** 1 Department of Health Sciences and Technology, ETH Zurich, Zurich, Switzerland; 2 Department of Internal Medicine and Vascular Medicine, Vivantes Hospital in Friedrichshain, Berlin, Germany

## Abstract

Platelets play a critical role in hemostasis. In addition to adhesion at the site of injury, phosphatidylserine (PS) exposing platelets (procoagulant) bind activated factor VIII (FVIIIa), facilitating coagulation. Effects of FVIIIa-platelet interactions on platelet activity are unclear. We explored how FVIIIa-platelet interactions affect their transition from a proaggregatory to procoagulant phenotype and how molecular modifications of recombinant FVIII (rFVIII) products might tune this phenotype shift. Platelets were collected from healthy donors and people with severe hemophilia A (HA) and were activated with thrombin and collagen-related peptide. Integrin αIIbβ3 activity and PS exposure were measured as markers of proaggregatory and procoagulant activities, respectively. Platelet phenotypes, rFVIIIa binding, and calcium influx were assessed by flow cytometry and confocal microscopy. rFVIIIa binding potentiated procoagulant activity while having no appreciable effect on percentage of platelets with activated integrin αIIbβ3. Potentiation of procoagulant activity was mediated by a thrombin-independent outside-in signaling cascade but involved integrin αIIbβ3. Similar trends were observed in platelets from healthy donors and patients with HA. A potential role of glycoprotein VI in rFVIIIa-platelet interactions was identified. rFVIIIa products with certain modifications to extend the circulation half-life of FVIII showed reduced binding to proaggregatory platelets compared to nonmodified rFVIIIa. Binding to procoagulant platelets was comparable across rFVIII products, except for rFVIII with site-specific PEGylation, which showed reduced binding. In conclusion, these results offer insights into FVIIIa-platelet interactions, and the potential impact of rFVIII modifications on platelet binding may inform clinical decision-making in HA.

## Key Points

• FVIII potentiates procoagulant platelet activity by thrombin-independent signaling involving integrin αIIbβ3 and glycoprotein VI.
• rFVIII products modified to extend the half-life show reduced binding to proaggregatory platelets.

## Visual Abstract

**Figure:**
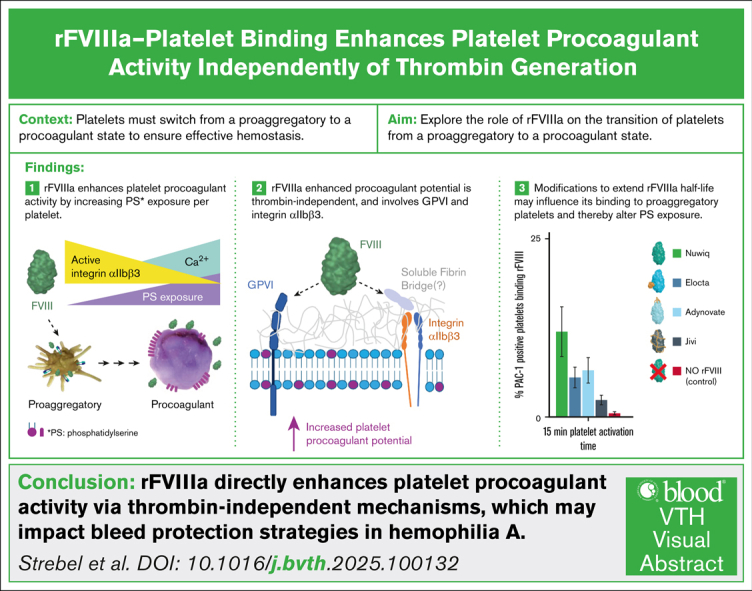

## Introduction

Platelets play a crucial role in coagulation by serving as key mediators in the formation of blood clots, which is essential for preventing excessive blood loss when vascular integrity is compromised.^1^, ^2^, ^3^ Upon vascular injury, platelets undergo activation and initiate primary hemostasis by adopting a proaggregatory phenotype. In this phenotype, platelets are highly adhesive and contractile, which ensures fast adherence and aggregation at the site of injury through interactions between platelet integrins and exposed basement membrane proteins.^4^ This process triggers the formation of a contracting platelet plug, which acts as the first physical barrier to stop bleeding.^5^, ^6^, ^7^

Simultaneously, a subset of platelets transitions from a proaggregatory to a procoagulant phenotype. The latter is characterized by low adherence to basement membrane proteins, low contractility, and increased exposure of phosphatidylserine (PS) on the outer platelet membrane.^8^^,^^9^ Surface exposure of PS plays an essential role in the procoagulant activity of platelets and the successive steps of tissue healing. It accelerates the coagulation cascade, thereby shaping and reinforcing the fibrin clot,^10^ while simultaneously serving as a signal to macrophages to clear aged/dead cells,^11^ promoting tissue regeneration.^12^ Clinically, decreased platelet surface PS has been reported to predict increased bleeding in patients with severe hemophilia A (HA).^13^

During blood clotting, interactions between platelets and coagulation factor VIII (FVIII) play a central role.^14^ A functional deficiency of FVIII in the circulation causes HA, a rare genetic bleeding disorder leading to uncontrolled and potentially life-threatening bleeding episodes.^15^^,^^16^ FVIII is transported in the bloodstream in a complex with von Willebrand factor (VWF) and dissociates from VWF at the site of injury after cleavage of FVIII by thrombin, which activates FVIII to activated FVIII (FVIIIa) and releases it from VWF.^17^, ^18^, ^19^ The dissociation releases VWF to promote platelet adhesion and aggregation (primary hemostasis) and FVIIIa to promote secondary hemostasis via the coagulation cascade.^18^, ^19^, ^20^ Upon activation of the coagulation cascade, PS exposed on the surface of platelets acts as a hub for the binding of FVIIIa and the formation of the tenase complex (FVIIIa and FIXa), leading to a localized boost of thrombin production through FX activation.^14^ Thrombin, in turn, plays dual roles: amplifying the activation of circulating platelets and promoting their aggregation; and inducing polymerization of the fibrin network that forms the clot.^10^^,^^21^

In addition to its well-established interactions with PS on the surface of procoagulant platelets, emerging research suggests that FVIIIa may also directly or indirectly interact with proaggregatory platelet receptors, such as glycoprotein VI (GPVI)^22^ and integrin αIIbβ3.^23^ This suggests a potential functional interaction between FVIIIa and platelets independent of intrinsic tenase complex formation and thrombin generation, which could affect platelet functions in the context of blood clotting. Consistent with this hypothesis, FVIII has been shown to affect platelet biomechanics, such as platelet spreading and aggregation.^24^

In this work, we performed dynamic flow cytometry measurements on platelets to assess how recombinant FVIIIa (rFVIIIa)–platelet interactions might regulate platelet functions, focusing on the phenotype shift from proaggregatory to procoagulant activity. This process, marked by PS exposure on the platelet membrane, is essential for the formation of tenase complexes and thrombin generation, which is pivotal for stabilizing fibrin clots and halting bleeding.^14^ By focusing on PS exposure on the platelet membrane, we aimed to evaluate the role of rFVIIIa in enhancing the blood coagulation efficiency of platelets.

## Methods

### Study design

Platelets were isolated from healthy human donors and from individuals with severe HA (FVIII coagulant activity [FVIII:C] <1 international unit [IU]/dL, <1%) as described in the supplemental Material. Washed platelets were used to eliminate confounding effects from other blood factors that interact with rFVIIIa (eg, VWF, FIXa, FX, and fibrinogen). Individuals with HA did not receive prophylactic rFVIII for at least 24 hours before blood sampling to ensure a low plasma FVIII (FVIII and rFVIII) level at the time of blood draws. The mean plasma FVIII level was 5.6% (standard deviation, 4.7%) on the day of blood draw.

Flow cytometry and confocal microscopy were used to evaluate the effects of rFVIIIa-platelet interactions on platelet functions, by focusing on the shift from a proaggregatory to a procoagulant phenotype upon platelet activation. The potential mechanisms underlying this effect were investigated by assessing intracellular calcium levels, the effects of thrombin, integrin αIIbβ3 and GPVI inhibition, and the binding of rFVIIIa to proaggregatory and procoagulant platelets over time. To assess whether modification of rFVIII (by Fc fusion or PEGylation) affects the binding of rFVIIIa to platelets and procoagulant activity, rFVIIIa-platelet binding experiments were conducted with different rFVIII products.

### rFVIII products

FVIIIa-platelet interactions were tested using 4 rFVIII products: simoctocog alfa (Nuwiq; Octapharma AG),^25^ efmoroctocog alfa (Elocta; Biogen Inc),^26^ rurioctocog alfa pegol (Adynovate; Takeda Pharmaceuticals Inc),^27^ and damoctocog alfa pegol (Jivi; Bayer AG).^28^

Nuwiq is a B-domain–deleted (BDD) rFVIII derived from human embryonic kidney cells with no chemical modification or protein fusion. Elocta is a BDD rFVIII derived from human embryonic kidney cells fused to a human immunoglobulin G1 Fc segment at the carboxyl terminus. Adynovate is a full-length rFVIII derived from Chinese hamster ovary cells, with 20 kDa polyethylene glycol (PEG) conjugation (PEGylation), 60% of which is located in the B domain. Jivi is a BDD rFVIII derived from baby hamster kidney cells, PEGylated at a cysteine in the A3 domain. All rFVIII products underwent buffer exchange using PD MiniTrap desalting columns with Sephadex G-25 into a buffer containing 4mM sodium citrate, 2mM CaCl_2_, 0.3M NaCl, 0.02% polysorbate 80%, and 1% human serum albumin at pH 7.3 and were stored at −80°C.^29^^,^^30^

### Quantification of the phenotype shift and binding of rFVIIIa to activated platelets

Approximately 300 × 10^3^/μL to 500 × 10^3^/μL isolated platelets were either fully activated using 1 IU/mL thrombin (Merck) and 5 μg/mL collagen-related peptide (CRP-XL; CambCol Laboratory) or selectively activated (only CRP-XL [5 μg/mL] or thrombin [1 IU/mL]) in Tyrode buffer containing 2.5mM CaCl_2_ and 1% human serum albumin. A total of 17.6nM various rFVIII products were included and thereby activated concomitantly to the activation of platelets. Because no preactivation step was used, there was no thrombin carryover from a separate activation step during our experiments. This incubation with thrombin ensures that rFVIII is in its active form (rFVIIIa) during platelet-binding assays.^22^^,^^29^, ^30^, ^31^ The concentration of 17.6nM was chosen considering the accumulation of FVIIIa upon the activated platelet membrane.^22^^,^^32^ Proaggregatory activity was assessed by measuring integrin αIIbβ3 activity using PAC-1,^33^ a monoclonal antibody that specifically recognizes activated αIIbβ3, coupled to fluorescein isothiocyanate (BD Biosciences; 1:50). Procoagulant activity was assessed by PS exposure measured by annexin V–brilliant violet (BV) 421 (Biolegend; 1:50), a protein that binds to the negatively charged membrane areas resulting from PS exposure.^10^^,^^34^ Throughout this study, we define procoagulant platelets as those exposing PS on their outer membrane surface. The terms procoagulant platelets, PS-exposing platelets, and platelets with a procoagulant phenotype are used interchangeably. In selected experiments, P-selectin expression was simultaneously assessed to further characterize the procoagulant phenotype by staining with phycoerythrin anti-human CD62P (BD Pharmingen; 1:10) concomitantly to activation.

Intracellular calcium levels were measured using 2μM Fluo-5N (Thermo Fisher) in dimethylsulfoxide.^34^ When integrin αIIbβ3 inhibition was required, platelets were treated with 3 or 20 μg/mL 10E5 (Novus Biologicals). GPVI was inhibited using 40 or 60 μg/mL glenzocimab (Acticor Biotech). Some experiments required thrombin-free conditions, and further details are provided in the supplemental Methods.

Binding of rFVIIIa to platelets over time was quantified using anti-FVIIIc mouse monoclonal antibody (PROGEN Biotechnik GmbH, Germany) conjugated to NHS AF647 (Thermo Scientific) or by direct labeling of the rFVIII products with NHS AF647 (Thermo Scientific) following the standard protocol.

Platelet activation was allowed to proceed for 15 to 105 minutes at room temperature, protected from light, and the reaction was halted by dilution. Immunofluorescence staining was measured on a Becton Dickinson Symphony A5 or a FACSLyric flow cytometer. To quantify the flow cytometry data, single platelets were gated according to the scheme described in supplemental Figure 1A and quantified by median fluorescence intensity or percentage of positive platelets, in which unstimulated platelets served as a negative control. Median fluorescence intensity was normalized to the highest value per fluorescent readout per experiment.

rFVIIIa-platelet interactions were also visualized using confocal microscopy. Proaggregatory platelets were fixed using 4% paraformaldehyde after 60 minutes of activation and immunostained for F-actin (Alexa Fluor 488). For procoagulant platelets, cells were live imaged, and PS exposure was quantified by annexin V staining (BV 421). In both cases, prelabeled (Alexa Fluor 647) rFVIIIa (Nuwiq) was added.

### Statistical analyses

Statistics and data visualization were performed using RStudio from Posit PBC. Statistical tests used are described further in the supplemental Methods. All data are expressed as mean ± standard error.

This study was conducted in accordance with the Declaration of Helsinki. Informed consent was obtained from all participants before blood samples were taken, and the participants’ privacy rights were observed. Institutional review board approval was received from ETH Zurich (Swiss Federal Institute of Technology).

## Results

### rFVIIIa enhances the procoagulant activity of activated platelets

**Figure 1.**
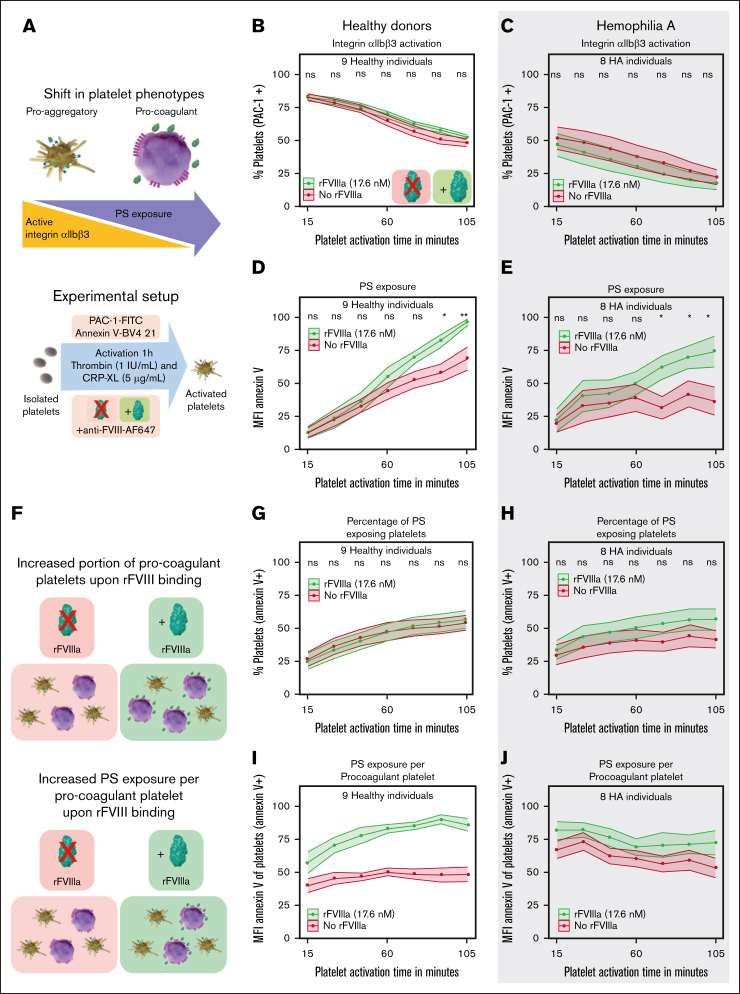
**Binding of rFVIIIa increases the procoagulant activity of platelets.** (A) Schematic representation of the methodology used for the quantification of rFVIIIa-platelet interactions and the corresponding shift from a proaggregatory to procoagulant phenotype. (B-C) Integrin αIIbβ3 activity was measured by percentage of PAC-1–fluorescein isothiocyanate (FITC) positive platelets from healthy donors (B) and patients with HA (C). (D-E) PS exposure was measured by the binding of fluorescently labeled annexin V–BV421 to platelets from healthy donors (D) and patients with HA (E). (F) Schematic representation of the phenotype shift induction in platelets stimulated with or without the presence of rFVIIIa; purple indicates PS-exposing procoagulant platelets, and yellow indicates proaggregatory platelets. (G-H) Percentage of PS-exposing platelets from healthy donors (G) and patients with HA (H) were measured with annexin V–BV421. (I-J) PS exposure per platelet was measured by the median fluorescence intensity (MFI) of the annexin V–BV421^+^ platelet population from healthy donors (I) and patients with HA (J). Data represent the mean ± standard error (SE) for platelets from 9 healthy donors and 8 patients with HA. MFI data were normalized to the highest value of each experiment. In panels D-E,I-J, data are normalized to the highest value per experiment and readout. Significance levels are indicated as follows: ∗*P* < .05; ∗∗*P* < .01; ∗∗∗*P* < .001; ∗∗∗∗*P* < .0001. All analyses and visualizations were done in RStudio. ns, not significant.

A consistent shift from a proaggregatory to a procoagulant phenotype was observed in both healthy and HA platelets upon activation. There was a decrease in proaggregatory platelets (integrin αIIbβ3 activity) and an increase in procoagulant platelets (PS exposure) over a time of 105 minutes (Figure 1A-E). Compared with activated platelets in the absence of rFVIIIa, the addition of rFVIIIa (17.6nM Nuwiq) did not significantly alter integrin αIIbβ3 activity (Figure 1B-C) but led to a significant increase in PS exposure (Figure 1D-E). This highlights a so far undescribed role of rFVIIIa in facilitating a procoagulant phenotype. This was further confirmed by the finding that most PS-positive platelets were also positive for P-selectin, supporting their classification as procoagulant and not apoptotic (supplemental Figure 1B-C).^35^ Importantly, even when focusing solely on the P-selectin–positive platelet population, rFVIIIa still increased PS exposure in platelets (supplemental Figure 1D).

The increased total PS exposure could have at least 2 explanations: either the total number of PS-exposing platelets is elevated, a higher number of PS molecules is exposed per platelet, or a combination of both (Figure 1F; supplemental Figure 1E). In healthy donors, the percentage of PS-exposing platelets remained very similar with and without rFVIIIa (Figure 1G), but the amount of PS exposed per platelet increased upon rFVIIIa addition (Figure 1I), indicating a potential increased capability of single platelets to form tenase complexes on their membrane. This effect was dose dependent, with 8.8nM rFVIIIa resulting in a modest increase in PS exposure per platelet, whereas 1nM rFVIIIa (corresponding to concentration of circulating nonactivated FVIII^29^) did not increase PS exposure per platelet compared to the control (supplemental Figure 1F-I). In HA platelets, the enhancement of PS exposure upon rFVIIIa addition was associated with a slight increase in both the number of PS-positive platelets as well as PS exposed per platelet (Figure 1H,J).

These results indicate that rFVIIIa enhances PS exposure in activated platelets, suggesting a role for FVIIIa in promoting the procoagulant phenotype and potentially improving hemostasis via increased thrombin generation through tenase complex formation.

### rFVIIIa supplementation increased platelet intracellular calcium levels

**Figure 2.**
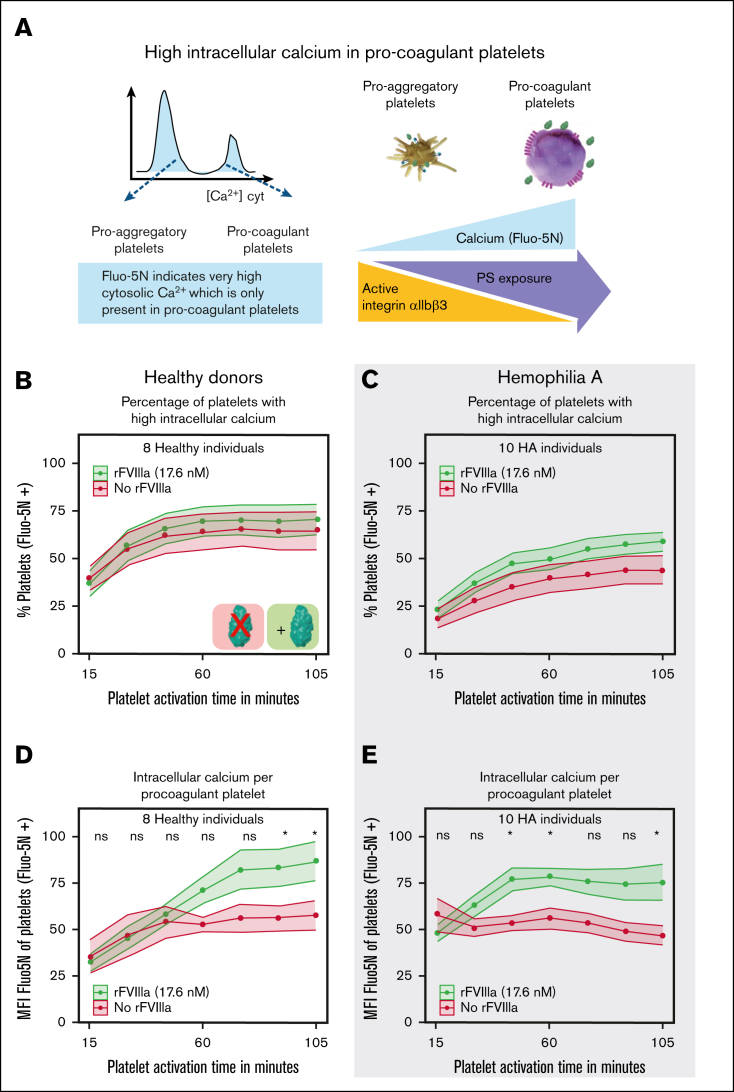
**Binding of rFVIIIa leads to increased intracellular calcium in healthy and HA platelets.** (A) Schematic representation of the methodology used for the quantification of supramaximal intracellular calcium measured with Fluo-5N–FITC. (B,C) The percentage of PS-exposing platelets with supramaximal intracellular calcium was measured using Fluo-5N–FITC labeling. (D-E) Intracellular calcium per PS-exposing platelet was measured by MFI of Fluo-5N–positive platelets. Data represent the mean ± SE for platelets from 8 healthy donors (B,D) and 10 patients with HA (C,E). MFI data were normalized to the highest value of each experiment. In panels D-E, data are normalized to the highest value per experiment and readout. Significance levels are indicated as follows: ∗*P* < .05; ∗∗*P* < .01; ∗∗∗*P* < .001; ∗∗∗∗*P* < .0001. All analyses and visualizations were done in RStudio.

The number of platelets with high calcium levels remained unchanged upon supplementation of rFVIIIa to healthy platelets (Figure 2A-B). In contrast, the calcium level per platelet increased significantly in the presence of rFVIIIa, with an increase of ∼50%, compared to an increase of ∼30% in the absence of rFVIIIa (Figure 2D). In HA platelets, both the number of calcium positive platelets as well as the calcium levels per platelet increased (Figure 2C,E), consistent with the findings on PS exposure.

Notably, a comparison of calcium levels in healthy and HA platelets revealed a more immediate response in HA platelets relative to controls (Figure 2D-E). This faster increase could be due to heightened cellular sensitivity to FVIII interactions, potentially functioning as an adaptive mechanism to improve the timing and effectiveness of hemostatic responses and thereby compensate for impaired coagulation.

### Enhancement of PS exposure by rFVIIIa is triggered by a thrombin-independent outside-in signaling mechanism

**Figure 3.**
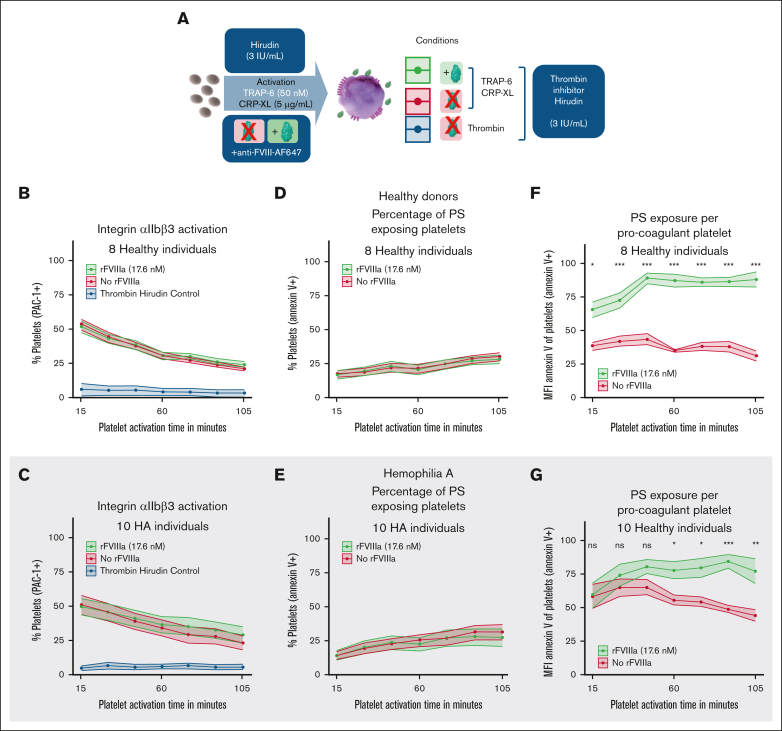
**Enhancement of PS exposure by rFVIIIa is thrombin independent.** (A) Schematic representation of the methodology used for the quantification of rFVIIIa-platelet interactions and the corresponding phenotype shift in the absence of thrombin. (B-C) The percentage of PAC-1–binding platelets was measured using PAC-1–FITC binding for healthy individuals (B) and patients with HA (C). (D-E) The percentage of PS-exposing platelets was measured based on annexin V–BV421 binding for healthy individuals (D) and patients with HA (E). (F-G) The amount of PS exposed per platelet was measured by MFI of annexin V–positive platelets for healthy individuals (F) and patients with HA (G). Data represent the mean ± SE of platelets from 8 healthy donors and 10 patients with HA. MFI data were normalized to the highest value of each experiment. Experiments were performed in the absence of thrombin. In panels F-G, data are normalized to the highest value per experiment and readout. Significance levels are indicated as follows: ∗*P* < .05; ∗∗*P* < .01; ∗∗∗*P* < .001; ∗∗∗∗*P* < .0001. All analyses and visualizations were done in RStudio.

Under thrombin-free conditions (presence of hirudin and use of thrombin receptor–activating peptide 6 [TRAP-6]; Figure 3A), the addition of rFVIIIa led to a significant increase in PS exposure per platelet, whereas integrin αIIbβ3 activity and the percentage of PS-exposing platelets remained unchanged (Figure 3B-G). Although residual thrombin activity cannot be entirely excluded, the observed effects in the presence of hirudin and absence of active thrombin support a direct contribution of rFVIIIa to platelet signaling.

### rFVIIIa binds to proaggregatory platelets

**Figure 4.**
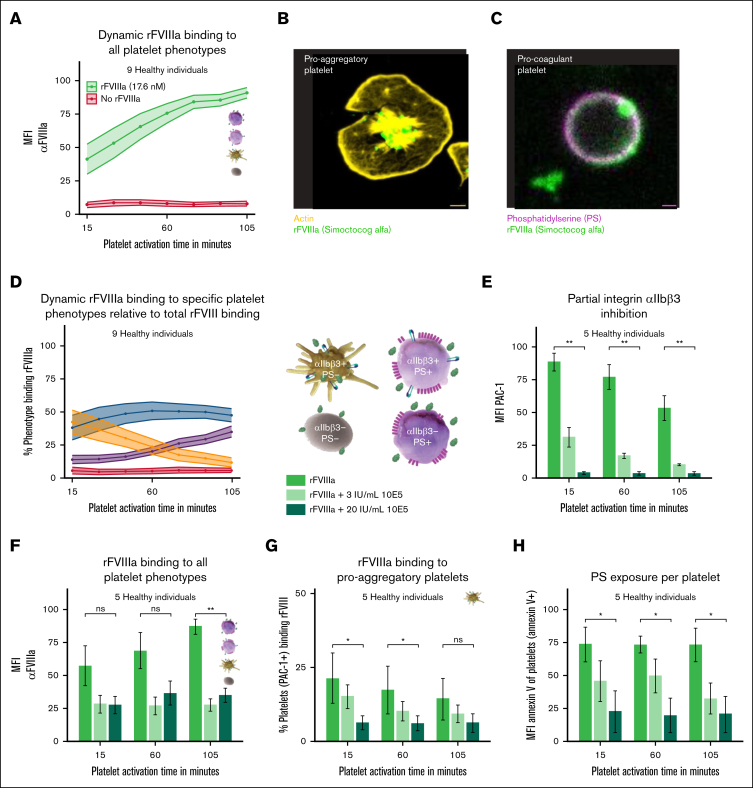
**Proaggregatory platelets and integrin αIIbβ3 are involved in mediating the enhanced platelet phenotype shift induced by rFVIIIa.** (A) Binding of rFVIIIa products to platelets was measured using anti-FVIII–AF647. (B-C) Confocal image of a proaggregatory platelet and a procoagulant platelet. Yellow indicates actin AF488; green, rFVIII-AF647; and purple, annexin V–BV421. (D) Percentage of platelet subtypes binding to rFVIIIa relative to all rFVIII-binding platelets: proaggregatory in yellow; double-positive in blue; procoagulant in purple; double negative in red. (E-H) Experiments were performed in the presence of 0, 3, or 20 μg/mL 10E5 and 17.6nM rFVIIIa; integrin αIIbβ3 activity was measured with the MFI of PAC-1–FITC (E); binding of rFVIIIa was measured using MFI of anti-FVIII–AF647 (F); percentage of proaggregatory (PAC-1–FITC positive) platelets binding rFVIII relative to all platelets (G); PS exposed per platelet was measured by MFI of annexin V–positive platelets (H). MFI data were normalized to the highest value of each experiment. Data represent the mean ± SE of platelets from 9 (panels A,C-D) or 5 healthy donors (panels E-H). In panels A,F,H, data are normalized to the highest value per experiment and readout. Significance levels are indicated as follows: ∗*P* < .05; ∗∗*P* < .01; ∗∗∗*P* < .001; ∗∗∗∗*P* < .0001. All analyses and visualizations were done in RStudio.

Although FVIII-platelet interactions are generally thought to occur via binding to PS exposed on procoagulant platelets, significant binding of rFVIIIa to platelets from healthy donors was detected as early as 15 minutes after platelet activation (Figure 4A), at which time the platelets exhibited high integrin αIIbβ3 activity and low levels of PS exposure (Figure 1B-E).

This was confirmed by confocal microscopy analysis, in which rFVIIIa binding to membranes of both platelet phenotypes was observed, only with differences in localization (Figure 4B-C). rFVIIIa was localized centrally in proximity to alpha granules of proaggregatory platelets (Figure 4B), while being more homogeneously distributed around the membrane of procoagulant platelets (Figure 4C).

Platelets were then categorized into 4 phenotypic groups: purely proaggregatory (only αIIbβ3 positive); procoagulant (only PS positive); double positive (αIIbβ3 and PS positive); and double negative (αIIbβ3 and PS negative; Figure 4D). In healthy platelets, almost 100% of PS-exposing platelets (procoagulant and double positive) bound rFVIIIa, as expected due to the high affinity of rFVIIIa for PS (supplemental Figure 2A,D). Approximately 25% of the proaggregatory (αIIbβ3-positive and PS-negative) platelets from healthy donors bound rFVIIIa (supplemental Figure 2A,D), indicative of a PS-independent mechanism that mediates its binding to proaggregatory platelets. Similar results were seen for platelets from patients with HA (supplemental Figure 2A,E).

We separately quantified how rFVIIIa binding was distributed among different platelet phenotypes over time (Figure 4D; supplemental Figure 2B,F). After 15 minutes of activation, almost 50% of rFVIIIa binding was mediated by proaggregatory platelets in healthy platelets (Figure 4D), highlighting a prominent role of this specific subset of platelets with active integrin αIIbβ3 that had a capacity to bind rFVIIIa. As activation progressed and platelets shifted phenotype, rFVIIIa binding shifted predominantly to procoagulant platelets (Figure 4D). In HA platelets, a significant but smaller fraction of αIIbβ3-positive platelets also bound rFVIIIa (supplemental Figure 2F). However, the proportion of proaggregatory platelets binding rFVIIIa (∼25%) was comparable between healthy and HA donors (supplemental Figure 2D-E).

The percentage of rFVIIIa bound to proaggregatory platelets decreased with time, whereas rFVIIIa bound to procoagulant platelets concurrently increased in both healthy and HA platelets, pointing to a potential transformation of FVIIIa-positive proaggregatory platelets into FVIIIa-positive procoagulant platelets over time (Figure 4D; supplemental Figure 2F). Unexpectedly, the fraction of double-positive platelets remained relatively constant over the entire time period, perhaps indicating that this population marks platelets in transition from a proaggregatory to a procoagulant phenotype.

In platelets from healthy donors, inhibition of integrin αIIbβ3 activity resulted in a significant dose-dependent reduction of αIIbβ3 activity (PAC-1 binding; Figure 4E; supplemental Figure 2H). Integrin αIIbβ3 inhibition correlated with decreased rFVIIIa binding to all platelet phenotypes (Figure 4F; supplemental Figure 2G). Focusing on phenotype-specific rFVIIIa binding, a dose-dependent decrease in rFVIIIa binding specifically to proaggregatory platelets upon αIIbβ3 inhibition was observed (Figure 4G). This reduction was accompanied by a dose-dependent reduction in PS exposure per platelet, suggesting that early interaction with proaggregatory platelets could influence subsequent PS exposure during coagulation (Figure 4H). Overall, rFVIIIa binding to all platelet phenotypes was significantly reduced at the low dose (3 μg/mL) of 10E5 (Figure 4F; supplemental Figure 2G). At higher concentrations of 10E5, a notable increase in the fraction of double-negative (αIIbβ3- and PS-negative) platelets that bind rFVIIIa was observed (supplemental Figure 2J-L).

These findings indicate that the early rFVIIIa binding is supported by activated integrin αIIbβ3 and likely contributes to the increased PS exposure during platelet activation.

### Involvement of GPVI in FVIII-platelet interactions and PS exposure

**Figure 5.**
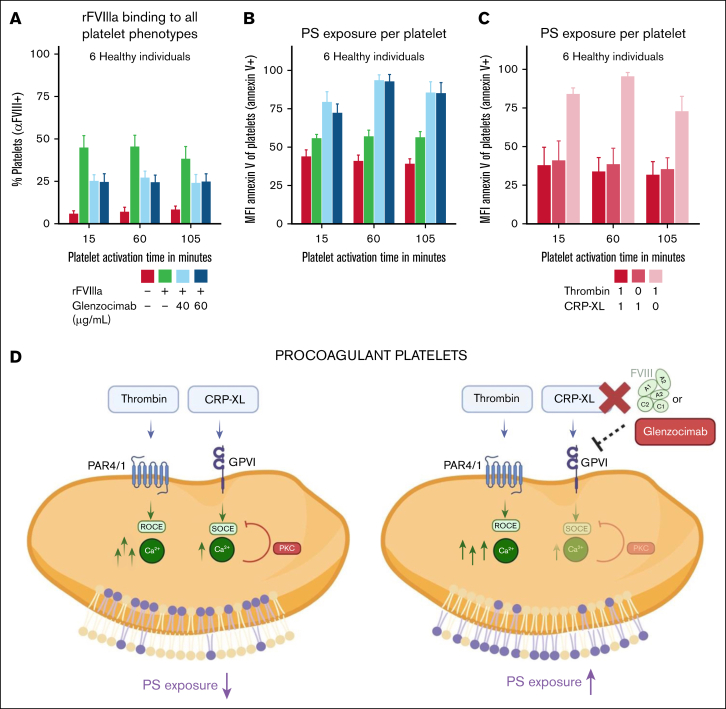
**Involvement of GPVI in FVIII-platelet interactions and PS exposure.** (A) Binding of rFVIIIa to platelets was measured in the presence of 0, 40, or 60 μg/mL glenzocimab using anti-FVIII–AF647. (B) PS exposure per platelet was quantified as MFI of annexin V in PS-positive platelets in the presence of 0, 40, or 60 μg/mL glenzocimab. (C) PS exposure per platelet was quantified as MFI of annexin V in PS-positive platelets in the presence of selective receptor stimulation with thrombin, CRP-XL, or both. (D) Schematic model summarizing the proposed mechanism: rFVIIIa interferes with GPVI engagement and shifts procoagulant signaling toward a thrombin-dominated pathway. Data were normalized to the highest value of each experiment. Results represent the mean ± SE of platelets (n = 6). Significance levels are indicated as follows: ∗*P* < .05; ∗∗*P* < .01; ∗∗∗*P* < .001; ∗∗∗∗*P* < .0001. All analyses and visualizations were performed in RStudio. PKC, protein kinase C; ROCE, receptor-operated calcium entry; SOCE, store-operated calcium entry.

To investigate the role of GPVI in rFVIIIa-platelet interactions, GPVI was inhibited using glenzocimab at 2 concentrations. GPVI inhibition led to a decrease in the percentage of rFVIIIa-binding platelets (Figure 5A) and, under sustained activation, a dose-dependent reduction in the amount of rFVIIIa bound per platelet (supplemental Figure 3A).

As expected, GPVI inhibition also reduced the overall proportion of PS-positive platelets (supplemental Figure 3B). However, PS exposure per PS-positive platelet increased significantly after glenzocimab treatment (Figure 5B). This mirrors the effect observed with rFVIIIa addition, although the percentage of PS-positive platelets remained unchanged with or without rFVIIIa.

To control for potential off-target or compensatory effects, we evaluated PS exposure over time under 3 different conditions: stimulation with thrombin alone, CRP-XL alone, or both combined. The highest PS exposure per platelet was observed under thrombin-only stimulation (Figure 5C), in which GPVI is not activated, consistent with the effects seen upon glenzocimab treatment. As expected, stimulation with a single agonist resulted in a lower percentage of PS-positive platelets (supplemental Figure 3C). These results support a role for GPVI in rFVIIIa-platelet binding and suggest that its modulation affects PS exposure per platelet, potentially via calcium signaling pathways (Figure 5D).

### Modifications of rFVIII products alter binding to platelets and modulate PS exposure

**Figure 6.**
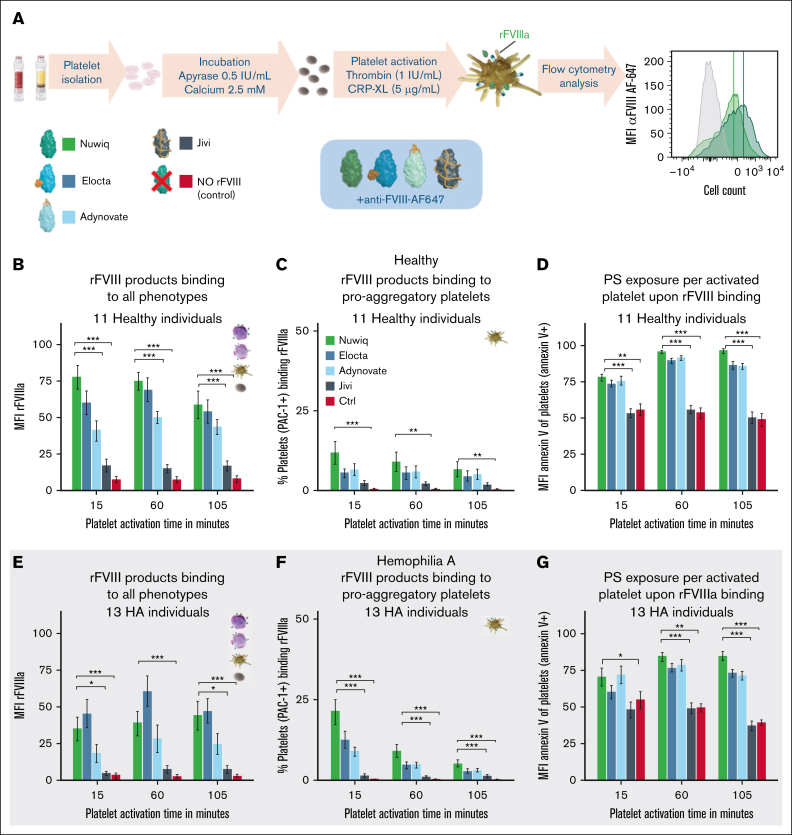
**Modulation of rFVIIIa binding to integrin αIIbβ3–active platelets influences the procoagulant activity of platelets.** (A) Schematic representation of the methodology used for the quantification of rFVIIIa-platelet interactions and the different rFVIII products used. (B,E) Binding of rFVIIIa to all phenotypes was quantified using the MFI of anti-FVIII–AF647. (C,F) Percentage of platelets showing active integrin αIIbβ3 and binding rFVIII with respect to all platelets. (D,G) PS exposed per platelet was measured by MFI annexin V of all annexin V–positive platelets. Data represent mean ± SE of platelets from 11 healthy donors (panels B-D) and from 13 patients with HA (panels E-G). MFI data were normalized to the highest value of each experiment. Experiments were performed in the presence of various rFVIIIa products. In panels B,D-E,G, data are normalized to highest value per experiment and readout. Significance levels are indicated as follows: ∗*P* < .05; ∗∗*P* < .01; ∗∗∗*P* < .001; ∗∗∗∗*P* < .0001. All analyses and visualizations were done in RStudio.

To further investigate the impact of rFVIIIa on the procoagulant platelet phenotype, interactions between healthy and HA platelets with different rFVIII products were examined. Nuwiq was used as the unmodified reference and compared with Elocta, Adynovate, and Jivi, which have been modified to extend their half-life (Figure 6A).

Jivi showed the lowest overall binding to platelets, particularly to proaggregatory platelets, compared to Nuwiq (Figure 6B-C,E-F). Adynovate displayed a similar reduction, although to a lesser extent. Nuwiq consistently demonstrated stronger binding to proaggregatory platelets and similar binding to double- and PS-positive phenotypes across all products, except Jivi (supplemental Figure 4E-F). These findings were consistent across both antibody-based and prelabeled detection methods (supplemental Figure 4G).

PS exposure per platelet increased over 15 to 60 minutes for all rFVIII products, with the exception of Jivi (Figure 6D,G). This effect was more marked when considering binding with proaggregatory platelets rather than with all platelet phenotypes (Figure 6B-D). In HA platelets, a clearer association was observed between PS exposure per platelet and the binding of rFVIIIa to proaggregatory platelets (Figure 6E-G). However, the differences remain subtle.

Among all rFVIII products tested, only Jivi exhibited reduced binding across all platelet phenotypes (supplemental Figure 4E-F) and significantly diminished procoagulant potential, as shown by lower PS exposure per procoagulant platelet (Figure 6D,G). No notable differences were observed among the rFVIII products regarding integrin αIIbβ3 activity or the percentage of PS-exposing platelets (supplemental Figure 4A,C-D).

## Discussion

Platelets play a dual role in hemostasis, driving primary hemostasis by adhering and aggregating at bleeding sites and modulating secondary hemostasis by binding FVIIIa via exposed PS to drive the coagulation cascade essential for effective clot formation and stabilization.^1^, ^2^, ^3^ However, the potential direct effects of FVIIIa on platelet functions, beyond its classical role in the coagulation cascade, remain underexplored. Here, we quantified early FVIIIa-platelet interactions to uncover their potential multifactorial impact on platelet function and coagulation kinetics. By deliberately excluding VWF, we specifically investigated direct interactions between free FVIIIa and activated platelets.

As expected, platelets displayed a temporal shift from a proaggregatory to procoagulant phenotype, with reduced integrin αIIbβ3 activity and increased PS exposure. Addition of rFVIIIa further increased PS exposure in platelets from both healthy donors and patients with HA. In healthy platelets, this effect was driven primarily by increased PS exposure per procoagulant platelet, whereas in HA platelets, both percentage of PS-exposing platelets and degree of PS exposure per platelet slightly increased. This attenuated response is consistent with reported data showing that platelets from patients with severe HA are in a preactivated state, which correlates with lower FVIII levels,^36^ and that platelet-dependent thrombin generation can partially compensate for FVIII deficiency.^35^

The concentration of 17.6nM rFVIIIa used in our experiments was based on estimates of local FVIIIa accumulation at sites of vascular injury. Indeed, although circulating FVIII levels average ∼1nM,^29^ several studies suggest that upon vascular damage and platelet activation, FVIII is rapidly converted to FVIIIa and concentrated at the platelet surface during activation.^32^^,^^37^ Notably, FVIIIa displays markedly increased affinity for PS compared with FVIII, with the dissociation constant decreasing from 5 ± 0.6nM to 0.65 ± 0.19nM,^37^ suggesting that local FVIIIa concentrations may reach the tens of nanomolar range, substantially exceeding plasma levels.^29^ The increase of PS exposure per platelet was dose dependent (supplemental Figure 1G). Notably, no increase in PS exposure per platelet was observed at 1nM rFVIIIa compared to control (supplemental Figure 1I), supporting the idea that functional effects of FVIIIa-platelet interactions likely require local FVIIIa concentrations exceeding those of nonactivated FVIII found in circulation.

Supporting the concept that rFVIIIa binding amplifies procoagulant platelet activity, our data show that rFVIIIa supplementation increased the intracellular calcium levels of platelets, consistent with the requirement for supramaximal intracellular calcium levels in procoagulant conversion.^8^^,^^34^^,^^38^ Approximately 95% of PS-positive platelets were also P-selectin positive and displayed increased PS exposure upon rFVIIIa treatment, confirming their procoagulant, rather than apoptotic, phenotype.^38^

Importantly, the rFVIIIa-mediated increase of platelet procoagulant activity is triggered by outside-in signaling that occurs independently of tenase complex formation and thrombin generation. Indeed, even when thrombin activity was inhibited by hirudin and platelet activation was driven by TRAP-6, rFVIIIa still increased PS exposure per platelet. Although further studies are needed to assess potential contributions from other coagulation factors (eg, FXa and FV), these results support a noncanonical signaling role of FVIIIa in platelet biology.

Beyond PS binding, our data suggest that FVIIIa interacts with proaggregatory platelet receptors. FVIIIa preferentially bound to platelets with high αIIbβ3 activity at early activation time points. Blocking αIIbβ3 reduced rFVIIIa binding and PS exposure, consistent with previous findings showing an eightfold increase in FVIIIa binding mediated by active αIIbβ3, possibly via fibrin bridging.^39^ Although the precise mechanism remains to be clarified, transient αIIbβ3 activation and associated signaling appear to facilitate FVIIIa-platelet interactions.

Similarly, GPVI played a nonredundant role. Inhibition of GPVI significantly reduced rFVIIIa binding to platelets. Interestingly, PS exposure per procoagulant platelet correlated inversely with GPVI activation, whereas overall platelet activation (percentage of PS-positive platelets) correlated positively. Here, we speculate that this may be explained by GPVI signaling restricting calcium influx through the STIM1–ORAI1 store-operated calcium entry axis via protein kinase C activation.^40^, ^41^, ^42^ Inhibiting GPVI could release this constraint, enhancing PS exposure per cell. This phenotype mirrors the effect of rFVIIIa supplementation, in which the percentage of PS-positive platelets remained constant, but each procoagulant platelet displayed higher PS exposure. These findings suggest that rFVIIIa may interfere with GPVI signaling, potentially shifting platelet activation away from a GPVI-dominated pathway toward a thrombin-driven program (Figure 5D). However, further mechanistic studies are needed to confirm this observation and to clarify whether FVIII also interacts with other platelet receptors that shape the procoagulant response.

Our comparative analysis revealed that structural modifications of rFVIII influence platelet interactions and procoagulant activity. Nuwiq exhibited stronger binding to proaggregatory platelets than some extended half-life rFVIII products, whereas binding to procoagulant platelets was comparable across products except for Jivi, which showed substantially reduced binding. The reduced binding observed with Elocta and Adynovate may result from structural modifications, such as Fc fusion or residual PEGylation, potentially interfering with platelet receptor interactions. Modifications targeting the C1/C2 domains are critical for both engagement of clearance receptors and platelet binding^28^^,^^43^ but may impair platelet interactions through steric hindrance or epitope masking, warranting further investigations. All in all, these findings highlight the importance of maximizing rFVIIIa-platelet interactions for optimal bleeding protection, because reduced binding was associated with decreased procoagulant activity.

Although our findings provide novel insights into the role of rFVIIIa in modulating platelet procoagulant activity, several limitations must be acknowledged. The in vitro nature of our experiments cannot fully capture the complexity of the hemostatic system in vivo, in which platelet-platelet and platelet-extracellular matrix interactions, shear stress, endothelial signaling, and the presence of other coagulation factors play crucial roles. Furthermore, the molecular mechanisms underlying rFVIIIa-platelet interactions remain incompletely characterized. Future studies using receptor-specific inhibitors, engineered variants, and epitope mapping will be required to delineate the contributions of αIIbβ3, GPVI, potential associated bridging molecules, and other receptors.

In conclusion, our study highlights previously underappreciated aspects of FVIIIa-platelet interactions, demonstrating that FVIIIa enhances platelet procoagulant activity through both PS-dependent binding and receptor-mediated pathways. These findings open new directions for exploring the noncanonical roles of FVIIIa in platelet biology and may have implications for HA management. Consideration of rFVIII products’ ability to modulate platelet procoagulant activity, in addition to pharmacokinetics, molecular stability, and immunogenicity, could refine therapeutic strategies for optimizing hemostatic efficacy.

Conflict-of-interest disclosure: All authors report research funding/travel grants from Octapharma AG.

## Supplementary Material

Supplemental Methods, Reference, and Figures
