# Supplementary material for: rFVIIIa-platelet binding enhances platelet procoagulant activity independently of thrombin generation
Source: Blood Vessel Thromb Hemost. 2025 Dec 10;3(1):100132. doi: 10.1016/j.bvth.2025.100132 (PMC12870833; doi:10.1016/j.bvth.2025.100132)
Supplement: Supplemental Methods, Reference, and Figures [file BVTH_VTH-2025-000373-mmc1.pdf]

rFVIIIa Enhances Platelet Procoagulant Activity Independent of Thrombin Generation

Anja Strebel[1], Sebastian Lickert[1], Robert Klamroth[2], Viola Vogel[1], Fabrizio A. Pennacchio[1]

[1]Department of Health Sciences and Technology, ETH Zurich, Switzerland.

[2]Department of Internal Medicine and Vascular Medicine, Vivantes Hospital in Friedrichshain, Berlin, Germany.

Supplemental Materials

1. Isolation of platelets

Platelets were isolated from healthy human donors and severe HA patients using 10 mL of drawn blood, collected in citrate tubes. The blood was centrifuged at 170 x *g* for 10 min at room temperature. From the resulting platelet-rich plasma, 1.6 mL was collected and mixed with 400 µL ACD solution (39mM citric acid, 75 mM sodium citrate, 135 mM dextrose, pH 7.4) and 1 µM of PGE_1 (Sigma Aldrich). This mixture was centrifuged at 900 x *g* for 5 min at room temperature, yielding a pellet that was subsequently resuspended in 1 mL Tyrode's buffer (10 mM sodium citrate, 150 mM NaCl, 2.9 mM KCl, 12 mM $NaHCO_3$, 0.34 mM Na_2HPO_4, 1 mM $MgCl_2$, 10 mM HEPES, 1 mM EDTA and 1% dextrose) with 200 µL ACD solution. The resuspended platelets underwent another round of washing and were resuspended in 1 mL Tyrode's buffer at pH 7.4. A 0.5 U/mL apyrase treatment (Sigma Aldrich) was applied to the platelet suspension for 30 min incubation.

2. Thrombin-free conditions

For experiments requiring thrombin-free conditions, 3 IU/mL hirudin (Sigma Aldrich), a potent inhibitor of thrombin[1], was added. Platelets were activated with 20 µM thrombin receptor activator for peptide 6 (TRAP-6) (Abcam, ab120801). rFVIII was activated to rFVIIIa prior to addition to the assays by incubating for 10 min with 22.5 IU/mL thrombin, then 22.5 IU/mL hirudin was added to neutralize the solution.

3. Statistical Tests

The datasets were first tested for normality using the Shapiro-Wilk test. If normality was confirmed, a T-test was used for comparing two groups, and a one-way analysis of variance (ANOVA) with Tukey's post-hoc test was applied for more than two groups. If the normality was not confirmed, the Wilcoxon test was used for comparing two groups, or the Friedman test was applied for repeated measures involving more than two groups, followed by Dunn’s multiple comparison test with Bonferroni correction.

References

1. Junren C, Xiaofang X, Huiqiong Z, et al. Pharmacological Activities and Mechanisms of Hirudin and Its Derivatives - A Review. *Front Pharmacol.Frontiers Media S.A.* 2021;12. doi:10.3389/fphar.2021.660757

SUPPLEMENTARY FIGURE 1 Binding of rFVIIIa increases the procoagulant activity of platelets. (A) On the left, the gating used on FlowJo is represented to filter for single platelets; to select the gate for rFVIIIa positive cells, unstimulated cells were used as negative control; on the right, example plot of intensity distribution after 60 min of stimulation and incubation of anti-FVIII-AF647 in platelets from a healthy donor. (B) Dotplot on Flowjo of single cell platelet population after 60 minutes of activation showing fluorescence intensity of αP-selectin antibody- PE vs. fluorescence intensity of annexin V-BV421 and the gating of the respective population being positive for PS in blue as well as the population being positive for PS and P-selectin in pink (C) The ratio of the percentage of platelets which stained positive for PS divided by the percentage of platelet which are positive for PS and P-selectin according to gates in B at every timepoint for the condition with (green) and without (red) addition of 17.6nM rFVIIIa. (D) Only considering P-selectin positive platelets the PS exposure was measured using the MFI of annexin V-BV421. MFI data were normalized to the highest value of each experiment, for the condition with (green) and without (red) addition of 17.6nM rFVIIIa for 6 healthy donors. (E) Color-coded histogram comparing the fluorescent distribution of annexin V-BV421 intensities after 60 min of activation in platelets from a healthy donor. The red curve represents the condition without rFVIII addition. (F) PS exposure was measured at 17.6nM (green), 8.8nM (lightgreen), and 0nM (red) rFVIII addition. The percentage of PS positive platelets were measured with annexin V-BV421. (G) The MFI of this PS positive population is shown. MFI data were normalized to the highest value of each experiment for 6 healthy donors. (H) PS exposure was measured at 17.6nM (green), 1nM (grey), and 0nM (red) rFVIII addition. The percentage of PS positive platelets were measured with annexin V-BV421. (I) The MFI of this PS positive population is shown. MFI data were normalized to the highest value of each experiment for 4 healthy donors. MFI, median fluorescence intensity; PS, phosphatidylserine; rFVIIIa, activated recombinant FVIII (Nuwiq). Significance levels are indicated as $p < 0.05$ (*), $p < 0.01$ (**), $p < 0.001$ (***), and $p < 0.0001$ (****). All analyses and visualizations were done in R Studio.

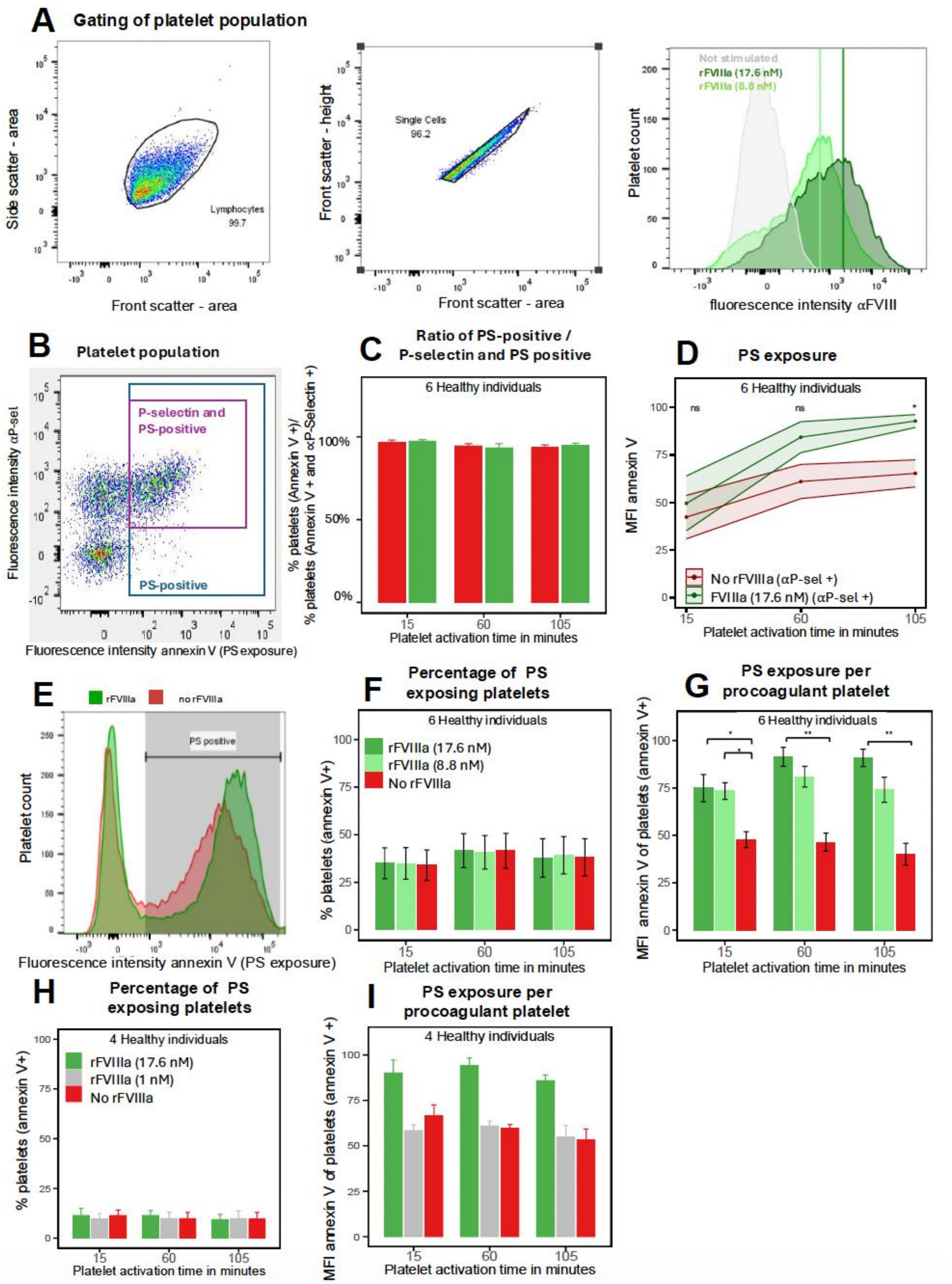
A
Gating of platelet population
Side scatter - area
Front scatter - area
Lymphocytes
99.7
Front scatter - height
Single Cells
96.2
Not stimulated
rFVIIIa (17.6 nM)
rFVIIIa (8.8 nM)
Platelet count
fluorescence intensity αFVIII
B
Platelet population
Fluorescence intensity αP-sel
P-selectin and
PS-positive
PS-positive
Fluorescence intensity annexin V (PS exposure)
C
Ratio of PS-positive /
P-selectin and PS positive
6 Healthy individuals
% platelets (Annexin V +)/
% platelets (Annexin V + and αP-Selectin +)
100%
50%
0%
15
60
105
Platelet activation time in minutes
D
PS exposure
6 Healthy individuals
MFI annexin V
ns
ns
*
No rFVIIIa (αP-sel +)
FVIIIa (17.6 nM) (αP-sel +)
Platelet activation time in minutes
E
rFVIIIa
no rFVIIIa
PS positive
Platelet count
Fluorescence intensity annexin V (PS exposure)
F
Percentage of PS
exposing platelets
6 Healthy individuals
rFVIIIa (17.6 nM)
rFVIIIa (8.8 nM)
No rFVIIIa
% platelets (annexin V+)
Platelet activation time in minutes
G
PS exposure per
procoagulant platelet
6 Healthy individuals
MFI annexin V of platelets (annexin V+)
Platelet activation time in minutes
H
Percentage of PS
exposing platelets
4 Healthy individuals
rFVIIIa (17.6 nM)
rFVIIIa (1 nM)
No rFVIIIa
% platelets (annexin V+)
Platelet activation time in minutes
I
PS exposure per
procoagulant platelet
4 Healthy individuals
MFI annexin V of platelets (annexin V +)
Platelet activation time in minutes

SUPPLEMENTARY FIGURE 2 Proaggregatory platelets and integrin αIIbβ3 are involved in mediating the enhanced platelet phenotype shift induced by rFVIIIa. (A) graphical illustration of gating for the percentage of specific platelet phenotype binding rFVIIIa. (B) graphical illustration of gating for quantifying the distribution of phenotypes when considering only rFVIIIa-binding platelets. (C) Graphical gating for each phenotype binding rFVIII in relation to all platelets. (D-E) Percentage of platelets per subtype binding to rFVIIIa relative to all platelets with this phenotype: proaggregatory in yellow; double-positive in blue; procoagulant in purple; resting in grey. In (D) healthy and (E) HA samples. (F) Distribution of phenotypes amongst rFVIIIa-binding platelets in HA samples (G) Percentage of platelets binding FVIII measured with anti-FVIII-AF647 (H) Percentage of platelets with active integrin αIIbβ3 measured with PAC-1 FITC (I) Percentage of platelets exposing PS measured with annexin V BV421, (J-L) Percentage of phenotypes binding rFVIII relative to all platelets for 0 μg/mL (J), 3 μg/mL (K), and 20 μg/mL (L) 10E5 , mean ± SD of 5 healthy donors with 0, 3 and 20 μg/mL of the integrin αIIbβ3 inhibitor and 17.6 nM rFVIIIa. HA, hemophilia A, MFI, median fluorescence intensity; PS, phosphatidylserine; rFVIIIa, activated recombinant factor VIII (Nuwiq); SE, standard error. Significance levels are indicated as $p < 0.05$ (*) and $p < 0.01$ (**). All analyses and visualizations were done in R Studio.

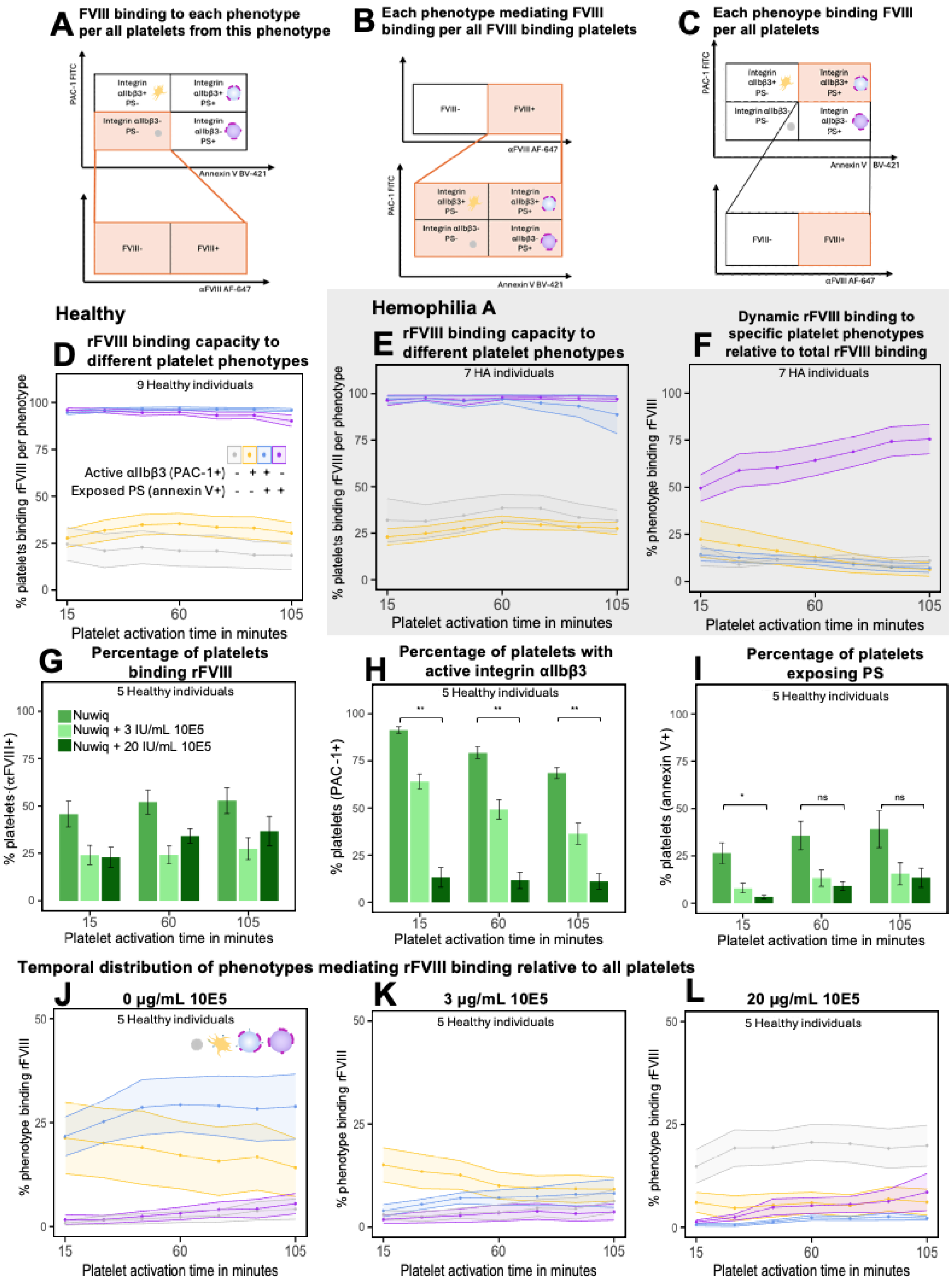

A FVIII binding to each phenotype per all platelets from this phenotype
B Each phenotype mediating FVIII binding per all FVIII binding platelets
C Each phenoype binding FVIII per all platelets
Healthy
D rFVIII binding capacity to different platelet phenotypes
9 Healthy individuals
Active αIIbβ3 (PAC-1+)
Exposed PS (annexin V+)
% platelets binding rFVIII per phenotype
Platelet activation time in minutes
Hemophilia A
E rFVIII binding capacity to different platelet phenotypes
7 HA individuals
F Dynamic rFVIII binding to specific platelet phenotypes relative to total rFVIII binding
% phenotype binding rFVIII
G Percentage of platelets binding rFVIII
5 Healthy individuals
Nuwiq
Nuwiq + 3 IU/mL 10E5
Nuwiq + 20 IU/mL 10E5
% platelets (αFVIII+)
H Percentage of platelets with active integrin αIIbβ3
% platelets (PAC-1+)
I Percentage of platelets exposing PS
% platelets (annexin V+)
Temporal distribution of phenotypes mediating rFVIII binding relative to all platelets
J 0 μg/mL 10E5
K 3 μg/mL 10E5
L 20 μg/mL 10E5

SUPPLEMENTARY FIGURE 3 Inhibition of GPVI alters rFVIIIa–platelet interactions and shifts procoagulant signaling. (A) Glenzocimab (40–60 μg/mL) decreased rFVIIIa binding per platelet in a dose-dependent manner, measured as MFI of anti-FVIII-AF647 among all rFVIII positive platelets. (B) Glenzocimab (40–60 μg/mL) reduced the percentage of PS positive platelets in a dose-dependent manner. (C) Agonist-specific stimulation showed that thrombin alone yielded fewer PS positive platelets compared to CRP-XL, while dual thrombin + CRP-XL stimulation produced the highest proportion of PS positive platelets. Data represent the mean ± SE of platelets from 6 healthy donors. MFI, median fluorescence intensity; PS, phosphatidylserine; rFVIIIa, activated recombinant factor VIII (Nuwiq); SE, standard error. All analyses and visualizations were performed in R Studio.

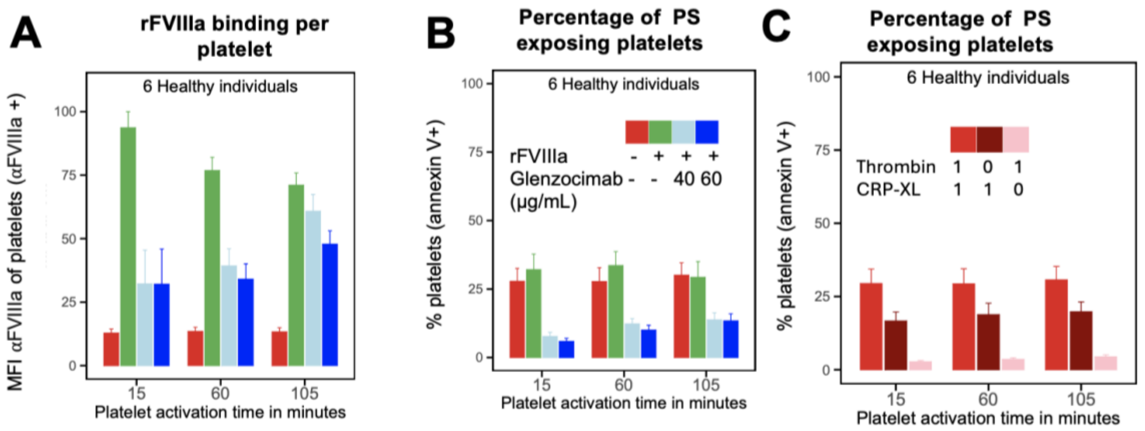

SUPPLEMENTARY FIGURE 4 Modulation of rFVIIIa binding to integrin αIIbβ3 active platelets influences their procoagulant activity. (A,C) Integrin αIIbβ3 activity was measured by percentage of PAC-1-FITC positive platelets, while (B, D) PS exposure was measured by percentage of annexin V-BV421 positive platelets from 11 healthy donors (A-B) and 13 HA patients (C-D). (E,F) Percentage of platelets per subtype binding to rFVIIIa relative to all platelets with this phenotype: proaggregatory in yellow; double-positive in blue; procoagulant in purple; resting in grey for 11 healthy (E) and 13 HA (F). (G) Binding of rFVIIIa to all phenotypes was quantified using the percentage of platelets binding prelabelled FVIII-AF647 with and without platelet stimulation; mean ± SE of platelets from 3-6 healthy donors using different rFVIII products; HA, hemophilia A; MFI, median fluorescence intensity; PS, phosphatidylserine; rFVIIIa, activated recombinant factor VIII. All analyses and visualizations were done in R Studio.

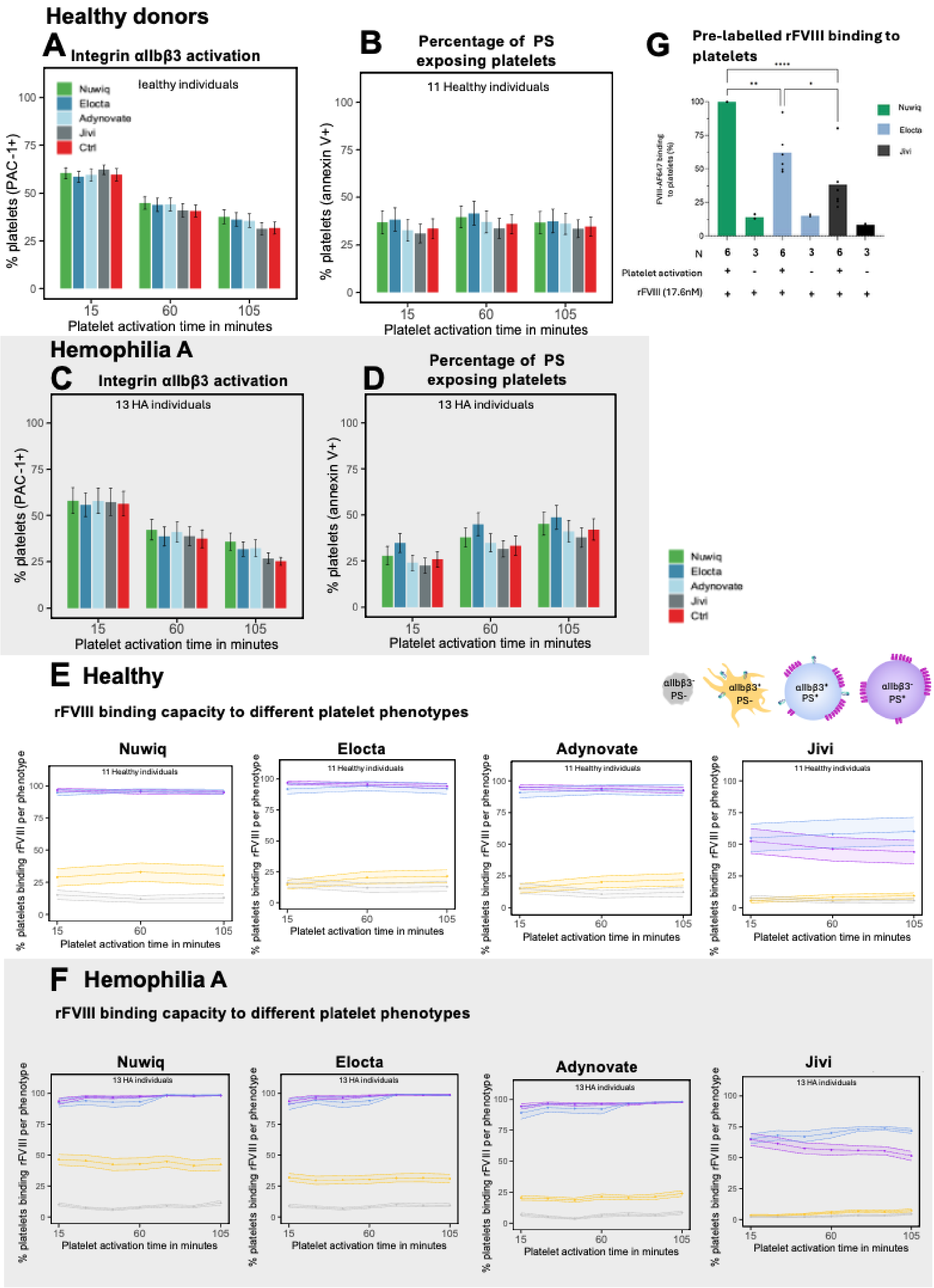

Healthy donors
A Integrin αIIbβ3 activation
lealthy individuals
Nuwiq
Elocta
Adynovate
Jivi
Ctrl
% platelets (PAC-1+)
Platelet activation time in minutes
B Percentage of PS exposing platelets
11 Healthy individuals
% platelets (annexin V+)
Platelet activation time in minutes
G Pre-labelled rFVIII binding to platelets
Platelet activation
rFVIII (17.6nM)
Hemophilia A
C Integrin αIIbβ3 activation
13 HA individuals
% platelets (PAC-1+)
Platelet activation time in minutes
D Percentage of PS exposing platelets
13 HA individuals
% platelets (annexin V+)
Platelet activation time in minutes
E Healthy
rFVIII binding capacity to different platelet phenotypes
Nuwiq
Elocta
Adynovate
Jivi
11 Healthy individuals
% platelets binding rFVIII per phenotype
Platelet activation time in minutes
F Hemophilia A
rFVIII binding capacity to different platelet phenotypes
13 HA individuals
% platelets binding rFVIII per phenotype
Platelet activation time in minutes

Supplementary Information Statistics P-values to Figure 1

B

	Product	Passed_Shapiro	pval
1	Ctrl	Pass	0.124223
2	Nuwiq	Pass	0.107868

	15	30	45	60	75	90	105
P value T test	0.9021	0.736	0.6976	0.3184	0.2894	0.1145	0.2372

C

	Product	Passed_Shapiro	pval
1	Ctrl	Fail	0.046833
2	Nuwiq	Fail	0.003013

	15	30	45	60	75	90	105
P value Wilcoxon	0.5737	0.5054	0.5737	0.7984	0.8785	0.5737	0.5054

D

	Product	Passed_Shapiro	pval
1	Ctrl	Pass	0.052397
2	Nuwiq	Fail	0.000831

	15	30	45	60	75	90	105
P value Wilcoxon	0.8252	0.7962	0.8633	0.2973	0.0939	0.0171	0.0083

E

	Product	Passed_Shapiro	pval
1	Ctrl	Fail	0.000324
2	Nuwiq	Fail	0.001787

	15	30	45	60	75	90	105
P value Wilcoxon	0.7984	0.5054	0.2933	0.5054	0.0281	0.0499	0.0204

G

	Product	Passed_Shapiro	pval
1	Ctrl	Fail	0.049039
2	Nuwiq	Fail	0.011759

	15	30	45	60	75	90	105
P value Wilcoxon	0.9314	0.6665	0.7962	0.9314	1	0.6665	0.6665

H

	Product	Passed_Shapiro	pval
1	Ctrl	Fail	0.000081
2	Nuwiq	Fail	0.022385

	15	30	45	60	75	90	105
P value Wilcoxon	0.7209	0.3282	0.4418	0.5054	0.4418	0.3823	0.2345

I

	Product	Passed_Shapiro	pval
1	Ctrl	Pass	0.098206
2	Nuwiq	Fail	0.000308

	15	30	45	60	75	90	105
P value Wilcoxon	0.0625	0.0106	0.0027	0	0	0.0011	0.002

J

	Product	Passed_Shapiro	pval
1	Ctrl	Pass	0.223368
2	Nuwiq	Fail	0.004459

	15	30	45	60	75	90	105
P value Wilcoxon	0.317	0.4936	0.1304	0.3823	0.1605	0.3282	0.2268

Supplementary Information Statistics P-values to Figure 2

B

	Product	Passed_Shapiro	pval
1	Ctrl	Fail	0.035151
2	Nuwiq	Pass	0.220082

	15	30	45	60	75	90	105
P value Wilcoxon	0.9372	0.6991	0.5887	0.4848	0.4848	0.5887	0.5887

C

	Product	Passed_Shapiro	pval
1	Ctrl	Fail	0.000099
2	Nuwiq	Pass	0.093330

	15	30	45	60	75	90	105
P value Wilcoxon	0.315	0.1903	0.1431	0.1051	0.0753	0.0753	0.0753

D

	Product	Passed_Shapiro	pval
1	Ctrl	Pass	0.926818
2	Nuwiq	Fail	0.003246

	15	30	45	60	75	90	105
P value Wilcoxon	0.8182	0.5887	0.4848	0.0931	0.0651	0.0411	0.045

E

	Product	Passed_Shapiro	pval
1	Ctrl	Pass	0.062706
2	Nuwiq	Fail	0.000175

	15	30	45	60	75	90	105
P value Wilcoxon	0.5787	0.1431	0.014	0.0185	0.0892	0.104	0.0443

Supplementary Information Statistics P-values to Figure 3

B

	Product	Passed_Shapiro	pval
1	Ctrl_H	Fail	0.005004
2	Nuwiq_H	Fail	0.003672

	15	30	45	60	75	90	105
P value Wilcoxon	0.7209	1	0.7984	0.5737	0.3823	0.4418	0.3823

C

	Product	Passed_Shapiro	pval
1	Ctrl_H	Fail	0.001852
2	Nuwiq_H	Fail	0.002681

	15	30	45	60	75	90	105
P value Wilcoxon	0.9118	0.9705	0.7959	0.7959	0.2973	0.3865	0.3865

D

	Product	Passed_Shapiro	pval
1	Ctrl_H	Pass	0.287731
2	Nuwiq_H	Pass	0.174920

	15	30	45	60	75	90	105
P value T test	0.8676	0.9316	0.8277	0.8747	0.9956	0.6987	0.6441

E

	Product	Passed_Shapiro	pval
1	Ctrl_H	Fail	0.011648
2	Nuwiq_H	Fail	0.000304

	15	30	45	60	75	90	105
P value Wilcoxon	0.7394	0.9705	0.9705	0.7959	0.8633	0.7962	0.7304

F

	Product	Passed_Shapiro	pval
1	Ctrl_H	Pass	0.060029
2	Nuwiq_H	Fail	0.000526

	15	30	45	60	75	90	105
P value Wilcoxon	0.0011	6e-04	9e-04	2e-04	2e-04	2e-04	9e-04

G

	Product	Passed_Shapiro	pval
1	Ctrl_H	Fail	0.003910
2	Nuwiq_H	Fail	0.000016

	15	30	45	60	75	90	105
P value Wilcoxon	0.9296	0.3865	0.0625	0.0142	0.0134	8e-04	0.0056

Supplementary Information Statistics P-values to Figure 4

A

	Product	Passed_Shapiro	pval
1	Ctrl	Fail	0.007453
2	Nuwiq	Fail	0.000007

	15	30	45	60	75	90	105
P value Wilcoxon	0.0106	8e-04	0	0	4e-04	0	4e-04

E

	Product	Passed_Shapiro	pval
1	Inhibitor_03	Fail	0.000022
2	Inhibitor_20	Fail	0.005531
3	Nuwiq	Fail	0.002400

	P value Friedman
15	0.0067
30	0.0067
45	0.0067
60	0.0067
75	0.0067

	Comparison	Dunn_TP_15	Dunn_TP_30	Dunn_TP_45	Dunn_TP_60	Dunn_TP_75	Dunn_TP_90	Dunn_TP_105
1	Inhibitor_03 - Inhibitor_20	0.230507737	0.231299615	0.231299615	0.231299615	0.231299615	0.231299615	0.231299615
2	Inhibitor_03 - Nuwiq	0.230507737	0.231299615	0.231299615	0.231299615	0.231299615	0.231299615	0.231299615
3	Inhibitor_20 - Nuwiq	0.001206331	0.001220856	0.001220856	0.001220856	0.001220856	0.001220856	0.001220856

F

	Product	Passed_Shapiro	pval
1	Inhibitor_03	Fail	0.040218
2	Inhibitor_20	Pass	0.116315
3	Nuwiq	Fail	0.000077

	P value Friedman
15	0.2466
30	0.0408
45	0.0067
60	0.0067
75	0.0067

	Comparison	Dunn_TP_15	Dunn_TP_30	Dunn_TP_45	Dunn_TP_60	Dunn_TP_75	Dunn_TP_90	Dunn_TP_105
1	Inhibitor_03 - Inhibitor_20	1.0000000	1.0000000	1.0000000	1.0000000	1.0000000	1.00000000	1.000000000
2	Inhibitor_03 - Nuwiq	0.6092754	0.2312996	0.1425489	0.0851318	0.1209149	0.00893840	0.007020491
3	Inhibitor_20 - Nuwiq	0.6092754	0.4718976	0.3107021	0.3116285	0.3593848	0.05887324	0.070581365

G

	Product	Passed_Shapiro	pval
1	Inhibitor_03	Fail	0.045388
2	Inhibitor_20	Fail	0.000310
3	Nuwiq	Fail	0.000453

	P value Friedman
15	0.015
30	0.015
45	0.015
60	0.015
75	0.0408

	Comparison	Dunn_TP_15	Dunn_TP_30	Dunn_TP_45	Dunn_TP_60	Dunn_TP_75	Dunn_TP_90	Dunn_TP_105
1	Inhibitor_03 - Inhibitor_20	0.4126917	0.3116285	0.6092754	0.8665331	1.0000000	1.0000000	1.0000000
2	Inhibitor_03 - Nuwiq	1.0000000	1.0000000	1.0000000	1.0000000	1.0000000	1.0000000	1.0000000
3	Inhibitor_20 - Nuwiq	0.2690581	0.1431446	0.2690581	0.4126917	0.6092754	0.7736971	0.7736971

H

	Product	Passed_Shapiro	pval
1	Inhibitor_03	Fail	0.000227
2	Inhibitor_20	Fail	0.000000
3	Nuwiq	Fail	0.010681

	P value Friedman
15	0.0743
30	0.0067
45	0.015
60	0.0224
75	0.015

	Comparison	Dunn_TP_15	Dunn_TP_30	Dunn_TP_45	Dunn_TP_60	Dunn_TP_75	Dunn_TP_90	Dunn_TP_105
1	Inhibitor_03 - Inhibitor_20	0.47078523	0.47189762	0.4126917	0.4126917	0.47189762	0.6879958	0.77369711
2	Inhibitor_03 - Nuwiq	0.96529911	0.68799583	0.6092754	0.8665331	0.68799583	0.9665964	0.35938479
3	Inhibitor_20 - Nuwiq	0.04834342	0.02666691	0.0174620	0.0327285	0.02666691	0.0851318	0.02162871

Supplementary Information Statistics P-values to Figure 5

A

	Product	Passed_Shapiro	pval
1	Ctrl	Fail	0.001382
2	Nuwiq	Fail	0.016446
3	Nuwiq_glen_40	Fail	0.009335
4	Nuwiq_glen_60	Fail	0.026651

	P value Friedman
15	0.0012
60	0.0056
105	0.0602

	Comparison	Dunn_TP_15	Dunn_TP_60	Dunn_TP_105
1	Ctrl - Nuwiq	0.003274651	0.001964483	0.1073536
2	Ctrl - Nuwiq_glen_40	0.311800721	0.518464398	1.0000000
3	Nuwiq - Nuwiq_glen_40	0.780439407	0.362334488	0.4346881
4	Ctrl - Nuwiq_glen_60	0.610341203	1.000000000	1.0000000
5	Nuwiq - Nuwiq_glen_60	0.411944788	0.107353606	0.7249163
6	Nuwiq_glen_40 - Nuwiq_glen_60	1.000000000	1.000000000	1.0000000

B

	Product	Passed_Shapiro	pval
1	Ctrl	Pass	0.465533
2	Nuwiq	Pass	0.096067
3	Nuwiq_glen_40	Fail	0.004435
4	Nuwiq_glen_60	Fail	0.029790

	P value Friedman
15	0.0081
60	9e-04
105	0.001

	Comparison	Dunn_TP_15	Dunn_TP_60	Dunn_TP_105
1	Ctrl - Nuwiq	1.000000000	1.000000000	0.724916319
2	Ctrl - Nuwiq_glen_40	0.003631034	0.004826324	0.005661869
3	Nuwiq - Nuwiq_glen_40	0.107353606	0.163909122	0.475076097
4	Ctrl - Nuwiq_glen_60	0.025600349	0.001409122	0.005661869
5	Nuwiq - Nuwiq_glen_60	0.434688081	0.067681490	0.475076097
6	Nuwiq_glen_40 - Nuwiq_glen_60	1.000000000	1.000000000	1.000000000

C

	Product	Passed_Shapiro	pval
1	Ctrl	Fail	0.000953
2	Ctrl_crp_only	Fail	0.000539
3	Ctrl_Tr_only	Fail	0.005101

	P value Friedman
15	0.0302
60	0.0025
105	0.0057

	Comparison	Dunn_TP_15	Dunn_TP_60	Dunn_TP_105
1	Ctrl - Ctrl_crp_only	1.00000000	1.000000000	1.00000000
2	Ctrl - Ctrl_Tr_only	0.03860472	0.003448228	0.05204407
3	Ctrl_crp_only - Ctrl_Tr_only	0.17523994	0.027892521	0.19796212

Supplementary Information Statistics P-values to Figure 6

B

	Product	Passed_Shapiro	pval
1	Adynovate	Pass	0.473894
2	Ctrl	Fail	0.000255
3	Elocta	Pass	0.303554
4	Jivi	Fail	0.022406
5	Nuwiq	Fail	0.003430

	P value Friedman
15	0
60	0
105	0

	Comparison	Dunn_TP_15	Dunn_TP_60	Dunn_TP_105
1	Adynovate - Ctrl	2.407675e-02	4.426770e-03	0.0019420883
2	Adynovate - Elocta	1.000000e+00	1.000000e+00	1.0000000000
3	Ctrl - Elocta	1.991011e-04	3.637467e-05	0.0002928861
4	Adynovate - Jivi	5.198247e-01	6.903395e-02	0.1145018796
5	Ctrl - Jivi	1.000000e+00	1.000000e+00	1.0000000000
6	Elocta - Jivi	1.501126e-02	1.338211e-03	0.0287171631
7	Adynovate - Nuwiq	3.724801e-01	1.000000e+00	1.0000000000
8	Ctrl - Nuwiq	3.092107e-06	5.248087e-06	0.0001056981
9	Elocta - Nuwiq	1.000000e+00	1.000000e+00	1.0000000000
10	Jivi - Nuwiq	5.665799e-04	2.608028e-04	0.0133943442

C

	Product	Passed_Shapiro	pval
1	Adynovate	Fail	0.000692
2	Ctrl	Fail	0.000001
3	Elocta	Fail	0.000052
4	Jivi	Fail	0.006701
5	Nuwiq	Fail	0.000065

	P value Friedman
15	0
60	0
105	0

	Comparison	Dunn_TP_15	Dunn_TP_60	Dunn_TP_105
1	Adynovate - Ctrl	0.0161086837	0.012213944	0.015011256
2	Adynovate - Elocta	1.0000000000	1.000000000	1.000000000
3	Ctrl - Elocta	0.0230776735	0.044008029	0.024613376
4	Adynovate - Jivi	1.0000000000	1.000000000	1.000000000
5	Ctrl - Jivi	1.0000000000	1.000000000	1.000000000
6	Elocta - Jivi	1.0000000000	1.000000000	1.000000000
7	Adynovate - Nuwiq	1.0000000000	1.000000000	1.000000000
8	Ctrl - Nuwiq	0.0003920967	0.001267471	0.002585583
9	Elocta - Nuwiq	1.0000000000	1.000000000	1.000000000
10	Jivi - Nuwiq	0.0983152107	0.253688940	0.360515652

D

	Product	Passed_Shapiro	pval
1	Adynovate	Pass	0.320783
2	Ctrl	Pass	0.169450
3	Elocta	Pass	0.266401
4	Jivi	Pass	0.162066
5	Nuwiq	Fail	0.000414

	P value Friedman
15	0
60	0
105	0

	Comparison	Dunn_TP_15	Dunn_TP_60	Dunn_TP_105
1	Adynovate - Ctrl	0.0215867070	5.213554e-04	1.274366e-02
2	Adynovate - Elocta	1.0000000000	1.000000e+00	1.000000e+00
3	Ctrl - Elocta	0.0563915213	5.135878e-03	6.542420e-03
4	Adynovate - Jivi	0.0028737628	1.336717e-03	2.199093e-02
5	Ctrl - Jivi	1.0000000000	1.000000e+00	1.000000e+00
6	Elocta - Jivi	0.0087788869	1.164973e-02	1.160901e-02
7	Adynovate - Nuwiq	1.0000000000	1.000000e+00	4.166466e-01
8	Ctrl - Nuwiq	0.0035265937	9.701556e-06	1.451997e-06
9	Elocta - Nuwiq	1.0000000000	1.000000e+00	6.424507e-01
10	Jivi - Nuwiq	0.0003594262	2.992271e-05	3.418285e-06

E

	Product	Passed_Shapiro	pval
1	Adynovate	Fail	0.000000
2	Ctrl	Fail	0.000000
3	Elocta	Fail	0.001581
4	Jivi	Fail	0.000000
5	Nuwiq	Fail	0.000011

	P value Friedman
15	0
60	0
105	0

	Comparison	Dunn_TP_15	Dunn_TP_30	Dunn_TP_45	Dunn_TP_60	Dunn_TP_75	Dunn_TP_90	Dunn_TP_105
1	Adynovate - Ctrl	5.406313e-02	7.182408e-02	0.2130189097	9.005428e-03	4.036742e-02	2.374027e-02	5.287218e-03
2	Adynovate - Elocta	5.525689e-01	2.130369e-01	0.1047527147	6.475023e-01	1.365580e-01	1.657071e-01	1.000000e+00
3	Ctrl - Elocta	2.619226e-05	6.012198e-06	0.0000116016	2.380537e-06	9.219671e-07	5.478144e-07	6.375445e-06
4	Adynovate - Jivi	1.000000e+00	1.000000e+00	1.0000000000	5.982951e-01	1.000000e+00	1.000000e+00	4.255731e-01
5	Ctrl - Jivi	1.000000e+00	1.000000e+00	1.0000000000	1.000000e+00	1.000000e+00	1.000000e+00	1.000000e+00
6	Elocta - Jivi	7.000662e-03	1.262293e-03	0.0047442634	1.922208e-03	7.068775e-04	5.384004e-04	3.974899e-03
7	Adynovate - Nuwiq	1.000000e+00	1.000000e+00	1.0000000000	1.000000e+00	1.000000e+00	1.000000e+00	1.000000e+00
8	Ctrl - Nuwiq	1.302607e-04	4.520028e-04	0.0014546289	4.076540e-04	1.988646e-04	4.190071e-04	4.001941e-05
9	Elocta - Nuwiq	1.000000e+00	1.000000e+00	1.0000000000	1.000000e+00	1.000000e+00	1.000000e+00	1.000000e+00
10	Jivi - Nuwiq	2.283862e-02	3.477596e-02	0.1505050002	7.692872e-02	3.747701e-02	6.934115e-02	1.505903e-02

F

1	Adynovate	Fail	6e-06
2	Ctrl	Fail	0e+00
3	Elocta	Fail	0e+00
4	Jivi	Fail	0e+00
5	Nuwiq	Fail	0e+00

	P value Friedman
15	0
60	0
105	0

	Comparison	Dunn_TP_15	Dunn_TP_30	Dunn_TP_45	Dunn_TP_60	Dunn_TP_75	Dunn_TP_90	Dunn_TP_105
1	Adynovate - Ctrl	1.408698e-03	5.710752e-04	5.967753e-04	3.918179e-05	5.155347e-05	8.012121e-05	4.876014e-05
2	Adynovate - Elocta	1.000000e+00	1.000000e+00	1.000000e+00	1.000000e+00	1.000000e+00	1.000000e+00	1.000000e+00
3	Ctrl - Elocta	1.523580e-04	4.783847e-04	3.495078e-04	1.453474e-04	9.432252e-05	1.234779e-04	6.097040e-04
4	Adynovate - Jivi	1.317583e-01	6.785914e-02	1.040835e-01	7.222070e-02	6.934897e-02	6.934897e-02	8.841113e-02
5	Ctrl - Jivi	1.000000e+00	1.000000e+00	1.000000e+00	5.369998e-01	6.311078e-01	7.757968e-01	5.094828e-01
6	Elocta - Jivi	2.721179e-02	5.983924e-02	7.222070e-02	1.611172e-01	1.012926e-01	9.148630e-02	3.967582e-01
7	Adynovate - Nuwiq	1.000000e+00	1.000000e+00	1.000000e+00	1.000000e+00	1.000000e+00	1.000000e+00	1.000000e+00
8	Ctrl - Nuwiq	1.032025e-06	7.749995e-07	2.269602e-06	8.693468e-07	1.254777e-07	2.311338e-07	1.537898e-06
9	Elocta - Nuwiq	1.000000e+00	1.000000e+00	1.000000e+00	1.000000e+00	1.000000e+00	1.000000e+00	1.000000e+00
10	Jivi - Nuwiq	6.514969e-04	5.001187e-04	1.963571e-03	6.196579e-03	1.262293e-03	1.323665e-03	9.805515e-03

G

	Product	Passed_Shapiro	pval
1	Adynovate	Fail	0.001134
2	Ctrl	Fail	0.031872
3	Elocta	Fail	0.009841
4	Jivi	Fail	0.037874
5	Nuwiq	Fail	0.000001

	P value Friedman
15	0
60	0
105	0

	Comparison	Dunn_TP_15	Dunn_TP_30	Dunn_TP_45	Dunn_TP_60	Dunn_TP_75	Dunn_TP_90	Dunn_TP_105
1	Adynovate - Ctrl	0.14573168	0.06812495	0.0016328632	1.675029e-03	4.956311e-03	3.977866e-03	6.156654e-03
2	Adynovate - Elocta	1.00000000	1.00000000	1.0000000000	1.000000e+00	1.000000e+00	1.000000e+00	1.000000e+00
3	Ctrl - Elocta	1.00000000	1.00000000	0.0310140836	3.042815e-03	3.721557e-03	7.794327e-04	1.598402e-03
4	Adynovate - Jivi	0.01110263	0.02420153	0.0009221302	1.926164e-03	4.540703e-03	2.112375e-03	3.640297e-03
5	Ctrl - Jivi	1.00000000	1.00000000	1.0000000000	1.000000e+00	1.000000e+00	1.000000e+00	1.000000e+00
6	Elocta - Jivi	0.85768741	0.69132585	0.0194975616	3.481414e-03	3.403778e-03	3.884007e-04	9.021017e-04
7	Adynovate - Nuwiq	1.00000000	1.00000000	1.0000000000	1.000000e+00	1.000000e+00	1.000000e+00	7.373060e-01
8	Ctrl - Nuwiq	0.20022029	0.07436169	0.0010655975	3.683357e-05	3.803137e-06	1.030622e-05	1.859120e-06
9	Elocta - Nuwiq	1.00000000	1.00000000	1.0000000000	1.000000e+00	1.000000e+00	1.000000e+00	1.000000e+00
10	Jivi - Nuwiq	0.01665969	0.02665176	0.0005933871	4.360214e-05	3.362020e-06	4.433492e-06	8.643305e-07

Supplementary Information Statistics P-values to Supplementary Figure 1

D

	Product	Passed_Shapiro	pval
1	Ctrl	Pass	0.497271
2	Nuwiq	Fail	0.002784

	15	60	105
P value Wilcoxon	0.5887	0.1275	0.0124

F

	Product	Passed_Shapiro	pval
1	Ctrl	Pass	0.202759
2	Nuwiq	Pass	0.213464
3	Nuwiq_05	Pass	0.197595

	15	60	105
ANOVA_P_Value	0.9954	0.9973	0.994
Tukey_Nuwiq-Ctrl	0.9959	0.9999	0.9997
Tukey_Nuwiq_05-Ctrl	0.9964	0.9983	0.9963
Tukey_Nuwiq_05-Nuwiq	1	0.9973	0.994

G

	Product	Passed_Shapiro	pval
1	Ctrl	Pass	0.138534
2	Nuwiq	Fail	0.010023
3	Nuwiq_05	Pass	0.769994

	Comparison	Dunn_TP_15	Dunn_TP_60	Dunn_TP_105
1	Ctrl - Nuwiq	0.0283325	0.003858442	0.002386919
2	Ctrl - Nuwiq_05	0.0283325	0.097789496	0.091377112
3	Nuwiq - Nuwiq_05	1.0000000	0.837711448	0.701858500

	P value Friedman
15	0.0421
60	0.0057
105	0.0057

H

	Product	Passed_Shapiro	pval
1	Ctrl	Fail	0.014043
2	Nuwiq	Fail	0.030771
3	Nuwiq_1nM	Fail	0.014449

	Comparison	Dunn_TP_15	Dunn_TP_60	Dunn_TP_105
1	Ctrl - Nuwiq	1	1	1
2	Ctrl - Nuwiq_1nM	1	1	1
3	Nuwiq - Nuwiq_1nM	1	1	1

	P value Friedman
15	0.1266
60	0.4724
105	0.7788

I

	Product	Passed_Shapiro	pval
1	Ctrl	Pass	0.165363
2	Nuwiq	Pass	0.156548
3	Nuwiq_1nM	Fail	0.033882

	Comparison	Dunn_TP_15	Dunn_TP_60	Dunn_TP_105
1	Ctrl - Nuwiq	0.34807492	0.04268439	0.04268439
2	Ctrl - Nuwiq_1nM	0.97785698	1.00000000	1.00000000
3	Nuwiq - Nuwiq_1nM	0.03194993	0.07233682	0.07233682

	P value Friedman
15	0.0388
60	0.0498
105	0.0388

Supplementary Information Statistics P-values to Supplementary Figure 2

G

	Product	Passed_Shapiro	pval
1	Inhibitor_03	Fail	0.012951
2	Inhibitor_20	Pass	0.185896
3	Nuwiq	Fail	0.000108

	P value Friedman
15	0.0224
30	0.0224
45	0.0067
60	0.0067
75	0.0067

	Comparison	Dunn_TP_15	Dunn_TP_30	Dunn_TP_45	Dunn_TP_60	Dunn_TP_75	Dunn_TP_90	Dunn_TP_105
1	Inhibitor_03 - Inhibitor_20	1.0000000	1.00000000	1.00000000	0.68799583	0.8665331	0.96659642	1.0000000
2	Inhibitor_03 - Nuwiq	0.1016846	0.05887324	0.02162871	0.02666691	0.0327285	0.04862862	0.1016846
3	Inhibitor_20 - Nuwiq	0.1016846	0.26905807	0.14314464	0.47189762	0.4126917	0.47189762	0.6092754

H

	Product	Passed_Shapiro	pval
1	Inhibitor_03	Pass	0.661750
2	Inhibitor_20	Fail	0.000621
3	Nuwiq	Pass	0.185065

	P value Friedman
15	0.0067
30	0.0067
45	0.0067
60	0.0067
75	0.0067

	Comparison	Dunn_TP_15	Dunn_TP_30	Dunn_TP_45	Dunn_TP_60	Dunn_TP_75	Dunn_TP_90	Dunn_TP_105
1	Inhibitor_03 - Inhibitor_20	0.231299615	0.231299615	0.231299615	0.231299615	0.231299615	0.231299615	0.31162847
2	Inhibitor_03 - Nuwiq	0.231299615	0.231299615	0.231299615	0.231299615	0.231299615	0.231299615	0.19797617
3	Inhibitor_20 - Nuwiq	0.001220856	0.001220856	0.001220856	0.001220856	0.001220856	0.001220856	0.00159174

I

	Product	Passed_Shapiro	pval
1	Inhibitor_03	Fail	0.019007
2	Inhibitor_20	Fail	0.000651
3	Nuwiq	Fail	0.009335

	P value Friedman
15	0.0067
30	0.0067
45	0.015
60	0.0224
75	0.0224

	Comparison	Dunn_TP_15	Dunn_TP_30	Dunn_TP_45	Dunn_TP_60	Dunn_TP_75	Dunn_TP_90	Dunn_TP_105
1	Inhibitor_03 - Inhibitor_20	0.6879958	0.471897621	1.00000000	1.0000000	1.0000000	1.0000000	1.0000000
2	Inhibitor_03 - Nuwiq	0.3116285	0.311628471	0.31162847	0.3116285	0.2312996	0.3593848	0.3116285
3	Inhibitor_20 - Nuwiq	0.0140332	0.007084179	0.04862862	0.0851318	0.1209149	0.1209149	0.1431446

Supplementary Information Statistics P-values to Supplementary Figure 3

A

	Product	Passed_Shapiro	pval
1	Ctrl	Pass	0.050942
2	Nuwiq	Fail	0.018519
3	Nuwiq_glen_40	Pass	0.137476
4	Nuwiq_glen_60	Fail	0.039389

	P value Friedman
15	0.0029
60	7e-04
105	0.0038

	Comparison	Dunn_TP_15	Dunn_TP_60	Dunn_TP_105
1	Ctrl - Nuwiq	0.0008495833	9.051479e-05	0.0006309813
2	Ctrl - Nuwiq_glen_40	0.5041016703	7.657805e-02	0.0197316858
3	Nuwiq - Nuwiq_glen_40	0.2265206099	3.971555e-01	1.0000000000
4	Ctrl - Nuwiq_glen_60	0.7374009116	3.971555e-01	0.2727317691
5	Nuwiq - Nuwiq_glen_60	0.1419446056	7.657805e-02	0.3623344879
6	Nuwiq_glen_40 - Nuwiq_glen_60	1.0000000000	1.000000e+00	1.0000000000

B

	Product	Passed_Shapiro	pval
1	Ctrl	Pass	0.085241
2	Nuwiq	Pass	0.104885
3	Nuwiq_glen_40	Pass	0.580885
4	Nuwiq_glen_60	Pass	0.529191

	15	60	105
ANOVA_P_Value	1e-04	5e-04	0.0079
Tukey_Nuwiq-Ctrl	0.8567	0.7074	0.9991
Tukey_Nuwiq_glen_40-Ctrl	0.0063	0.0446	0.0464
Tukey_Nuwiq_glen_60-Ctrl	0.003	0.0184	0.0408
Tukey_Nuwiq_glen_40-Nuwiq	0.001	0.0042	0.061
Tukey_Nuwiq_glen_60-Nuwiq	5e-04	0.0016	0.0538
Tukey_Nuwiq_glen_60-Nuwiq_glen_40	0.9868	0.9751	0.9999

C

	Product	Passed_Shapiro	pval
1	Ctrl	Fail	0.009727
2	Ctrl_crp_only	Fail	0.031621
3	Ctrl_Tr_only	Pass	0.699807

	P value Friedman
15	0.0025
60	0.0025
105	0.0025

	Comparison	Dunn_TP_15	Dunn_TP_60	Dunn_TP_105
1	Ctrl - Ctrl_crp_only	0.838456105	0.838456105	0.838456105
2	Ctrl - Ctrl_Tr_only	0.001616177	0.001616177	0.001616177
3	Ctrl_crp_only - Ctrl_Tr_only	0.052044070	0.052044070	0.052044070

Supplementary Information Statistics P-values to Supplementary Figure 4

A

	Product	Passed_Shapiro	pval
1	Adynovate	Pass	0.312317
2	Ctrl	Pass	0.514882
3	Elocta	Pass	0.638242
4	Jivi	Pass	0.531823
5	Nuwiq	Pass	0.295057

	15	60	105
ANOVA_P_Value	0.9282	0.8498	0.614
Tukey_Ctrl-Adynovate	1	0.9476	0.9317
Tukey_Elocta-Adynovate	0.9994	1	0.9999
Tukey_Jivi-Adynovate	0.9664	0.9619	0.9044
Tukey_Nuwiq-Adynovate	0.9994	0.9999	0.993
Tukey_Elocta-Ctrl	0.999	0.9582	0.8858
Tukey_Jivi-Ctrl	0.9715	1	1
Tukey_Nuwiq-Ctrl	0.9996	0.8991	0.742
Tukey_Jivi-Elocta	0.9047	0.9705	0.8501
Tukey_Nuwiq-Elocta	0.9907	0.9996	0.9984
Tukey_Nuwiq-Jivi	0.9934	0.9211	0.6924

B

	Product	Passed_Shapiro	pval
1	Adynovate	Pass	0.517295
2	Ctrl	Pass	0.756464
3	Elocta	Pass	0.537528
4	Jivi	Pass	0.769787
5	Nuwiq	Pass	0.291262

	15	60	105
ANOVA_P_Value	0.8813	0.8737	0.9858
Tukey_Ctrl-Adynovate	1	0.9999	0.9997
Tukey_Elocta-Adynovate	0.9508	0.9788	0.9997
Tukey_Jivi-Adynovate	0.9996	0.993	0.9972
Tukey_Nuwiq-Adynovate	0.9846	0.9973	1
Tukey_Elocta-Ctrl	0.9735	0.955	0.9955
Tukey_Jivi-Ctrl	0.9976	0.9983	0.9999
Tukey_Nuwiq-Ctrl	0.9939	0.9903	0.9988
Tukey_Jivi-Elocta	0.8842	0.8564	0.9849
Tukey_Nuwiq-Elocta	0.9996	0.9992	1
Tukey_Nuwiq-Jivi	0.948	0.9421	0.9938

C

	Product	Passed_Shapiro	pval
1	Adynovate	Fail	0.009389
2	Ctrl	Fail	0.001148
3	Elocta	Fail	0.011008
4	Jivi	Fail	0.011006
5	Nuwiq	Fail	0.043164

	P value Friedman
15	0.1879
30	0.3697
45	0.2109
60	0.0647
75	8e-04

	Comparison	Dunn_TP_15	Dunn_TP_30	Dunn_TP_45	Dunn_TP_60	Dunn_TP_75	Dunn_TP_90	Dunn_TP_105
1	Adynovate - Ctrl	1	1	1	1	1	1	1.0000000
2	Adynovate - Elocta	1	1	1	1	1	1	1.0000000
3	Ctrl - Elocta	1	1	1	1	1	1	1.0000000
4	Adynovate - Jivi	1	1	1	1	1	1	1.0000000
5	Ctrl - Jivi	1	1	1	1	1	1	1.0000000
6	Elocta - Jivi	1	1	1	1	1	1	1.0000000
7	Adynovate - Nuwiq	1	1	1	1	1	1	1.0000000
8	Ctrl - Nuwiq	1	1	1	1	1	1	0.5986502
9	Elocta - Nuwiq	1	1	1	1	1	1	1.0000000
10	Jivi - Nuwiq	1	1	1	1	1	1	1.0000000

D

	Product	Passed_Shapiro	pval
1	Adynovate	Fail	0.012217
2	Ctrl	Fail	0.013448
3	Elocta	Fail	0.007622
4	Jivi	Fail	0.031860
5	Nuwiq	Fail	0.002045

	P value Friedman
15	3e-04
30	0
45	1e-04
60	0
75	0

	Comparison	Dunn_TP_15	Dunn_TP_30	Dunn_TP_45	Dunn_TP_60	Dunn_TP_75	Dunn_TP_90	Dunn_TP_105
1	Adynovate - Ctrl	1.0000000	1.0000000	1.0000000	1.0000000	1.0000000	1.0000000	1
2	Adynovate - Elocta	1.0000000	0.6189949	0.6046503	1.0000000	0.7956473	1.0000000	1
3	Ctrl - Elocta	1.0000000	1.0000000	1.0000000	1.0000000	1.0000000	1.0000000	1
4	Adynovate - Jivi	1.0000000	1.0000000	1.0000000	1.0000000	1.0000000	1.0000000	1
5	Ctrl - Jivi	1.0000000	1.0000000	1.0000000	1.0000000	1.0000000	1.0000000	1
6	Elocta - Jivi	0.7357581	0.3017300	0.8141272	0.6792172	0.3539061	0.9464262	1
7	Adynovate - Nuwiq	1.0000000	1.0000000	1.0000000	1.0000000	1.0000000	1.0000000	1
8	Ctrl - Nuwiq	1.0000000	1.0000000	1.0000000	1.0000000	1.0000000	1.0000000	1
9	Elocta - Nuwiq	1.0000000	1.0000000	1.0000000	1.0000000	1.0000000	1.0000000	1
10	Jivi - Nuwiq	1.0000000	1.0000000	1.0000000	1.0000000	1.0000000	1.0000000	1

G

	Product	Passed_Shapiro	pval
1	Ctrl	Pass	0.000000
2	Nuwiq	Pass	0.331698
3	Nuwiq_1nM	Pass	0.280983

P value Friedman

0.00247875217666636

Dunns multiple comparisons

	group1	group2	p.adj	p.adj.signif
1	Damocotogalfapegol_AF647_SP	Efmorocotocogalfa_AF647_SP	0.1521384167	ns
2	Damocotogalfapegol_AF647_SP	Simoctocogalfa_AF647_SP	0.0006722268	***
3	Efmorocotocogalfa_AF647_SP	Simoctocogalfa_AF647_SP	0.0478684273	*
